# Mutant RAS-driven Secretome Causes Skeletal Muscle Defects in Breast Cancer

**DOI:** 10.1158/2767-9764.CRC-24-0045

**Published:** 2024-05-15

**Authors:** Ruizhong Wang, Aditi S. Khatpe, Brijesh Kumar, Henry Elmer Mang, Katie Batic, Adedeji K. Adebayo, Harikrishna Nakshatri

**Affiliations:** 1 Department of Surgery, Indiana University School of Medicine, Indianapolis, Indiana.; 2 Department of Biochemistry and Molecular Biology, Indiana University School of Medicine, Indianapolis, Indiana.; 3 Richard L Roudebush VA Medical Center, Indianapolis, Indiana.

## Abstract

Cancer-induced skeletal muscle defects differ in severity between individuals with the same cancer type. Cancer subtype-specific genomic aberrations are suggested to mediate these differences, but experimental validation studies are very limited. We utilized three different breast cancer patient-derived xenograft (PDX) models to correlate cancer subtype with skeletal muscle defects. PDXs were derived from brain metastasis of triple-negative breast cancer (TNBC), estrogen receptor–positive/progesterone receptor–positive (ER+/PR+) primary breast cancer from a BRCA2-mutation carrier, and pleural effusion from an ER+/PR− breast cancer. While impaired skeletal muscle function as measured through rotarod performance and reduced levels of circulating and/or skeletal muscle *miR-486* were common across all three PDXs, only TNBC-derived PDX activated phospho-p38 in skeletal muscle. To further extend these results, we generated transformed variants of human primary breast epithelial cells from healthy donors using *HRAS^G12V^* or *PIK3CA^H1047R^* mutant oncogenes. Mutations in *RAS* oncogene or its modulators are found in approximately 37% of metastatic breast cancers, which is often associated with skeletal muscle defects. Although cells transformed with both oncogenes generated adenocarcinomas in NSG mice, only HRAS^G12V^-derived tumors caused skeletal muscle defects affecting rotarod performance, skeletal muscle contraction force, and *miR-486, Pax7*, pAKT, and *p53* levels in skeletal muscle. Circulating levels of the chemokine CXCL1 were elevated only in animals with tumors containing *HRAS^G12V^* mutation. Because RAS pathway aberrations are found in 19% of cancers, evaluating skeletal muscle defects in the context of genomic aberrations in cancers, particularly RAS pathway mutations, may accelerate development of therapeutic modalities to overcome cancer-induced systemic effects.

**Significance:**

Mutant RAS- and PIK3CA-driven breast cancers distinctly affect the function of skeletal muscle. Therefore, research and therapeutic targeting of cancer-induced systemic effects need to take aberrant cancer genome into consideration.

## Introduction

Skeletal muscle dysfunction affects the quality of life of many patients with breast cancer, usually due to role limitations associated with poor physical functioning and body pain. About 39% of patients with breast cancer experience skeletal muscle defects that contribute to mortality in these patients (1). Clinical data have shown that age, body composition, disease stage, and progression contribute to breast cancer–associated skeletal muscle dysfunction (1–3). However, there is very limited knowledge regarding how breast cancer subtypes affect skeletal muscle function, although it is believed that aggressive tumor types have greater impact on skeletal muscles of patients (4).

Recent preclinical studies by us and others have demonstrated that different subtypes of breast cancer have distinct physical and molecular impacts on skeletal muscles of animals. We had used the FVB/N mouse mammary tumor virus-polyoma middle tumor antigen (MMTV-PyMT) model, which represents the “luminal B” subtype of human breast cancer based on gene expression patterns in relation to human breast cancers but initiates as an estrogen receptor–positive (ER+) tumor but eventually progresses into ER-negative tumor (5, 6). In this model, animals had early mammary tumor occurrence (∼10 weeks post birth) and fast tumor growth rate, which was accompanied with quick reduction of grip strength, rotarod performance, and muscle contraction forces (7). By contrast, in the FVB/N MMTV-Neu (Neu+) model, which represents the HER2-amplified subtype of human breast cancer (8), animals had late mammary tumor occurrence (∼20 weeks after birth) and slower tumor growth rate, which was accompanied with mild reduction of grip strength, rotarod performance, and muscle contraction force (9). Reduced body fat, muscle fiber size, muscle mitochondria content, and increased body free water were observed in PyMT+ mice, but not in Neu+ mice (7, 9). Furthermore, profound downregulation of *miR-486*, *Myod,* and *Pax7* occurred in skeletal muscles of PyMT+ mice, but not in Neu+ mice (7, 9). Other differential changes were observed in circulating cytokines, muscle stem cell surface markers, skeletal muscle gene expression, and protein levels between PyMT+ mice and Neu+ mice (7, 9). Because of limited clinical data, however, whether functional and molecular defects occur differentially in skeletal muscles of patients with different breast cancer subtypes remain unclear. Therefore, there is an unmet need to dissect the impact of breast cancer subtypes on human skeletal muscle function.

Patient-derived xenograft (PDX) models have been shown to recapitulate some of the characteristics of human cancer by retaining the genomic features of tumors across different stages, subtypes, and diverse treatment regimens (10). In breast cancer, PDX models have been shown to be reliable in preclinical research and in exploring drug resistance mechanisms (10, 11). In the current study, we established three PDX models representing different breast cancer subtypes to study cancer-associated skeletal muscle dysfunction. We found that PDX derived from brain metastasis of a triple-negative breast cancer (TNBC) caused the most severe skeletal muscle dysfunction along with loss of *miR-486*, myogenesis regulators (i.e., *Pax7* and *MyoD*), and activation of p-38 MAPK pathway in skeletal muscle. These skeletal muscle changes were less apparent in mice with ER+ and progesterone receptor (PR) positive (ER+/PR+) or ER+/PR− PDXs. To determine which among the genomic aberrations found in primary and/or metastatic breast cancers can recapitulate the observations in PDX mice, we established another model in which primary breast tumors were established via transformation of human breast epithelial cells with *HRAS^G12V^* or *PIK3CA^H1047R^* mutants. Although RAS pathway mutations in primary breast cancer is approximately 2% (12), a recent study involving genomic analyses of 379 metastatic samples from 301 patients found 40%, 24%, 18%, and 12% of metastasis harboring aberrations in *H-RAS*, *K-RAS*, *M-RAS,* and *N-RAS* genes, respectively (13). We observed profound skeletal muscle defects in mice harboring mutant HRAS-driven tumors compared with those with mutant PIK3CA-driven tumors, despite both tumors being adenocarcinomas. These results suggest that breast cancer subtype uniquely impacts skeletal muscle function and, moving forward, it is critical to consider RAS pathway mutations not only in breast cancer therapy but also in breast cancer–induced skeletal muscle defects.

## Materials and Methods

### PDXs and Oncogene Transformed Human Cells in Culture

PDXs were derived in the lab. One PDX was derived from brain metastasis of a TNBC. The second PDX was derived from pleural effusion of an ER+/PR− breast cancer. The third is an ER+PR+ primary breast tumor from a BRCA2 germline mutation carrier. While PDXs from TNBC and ER+/PR+ cases were directly from intact tumors with associated tumor microenvironment, ER+/PR− PDX was generated from cells collected from pleural effusion. Briefly, pleural effusion was spun down, washed in PBS and cell pellet resuspended in Hank's Balanced Salt Solution was implanted into the mammary fat pad of NOD scid gamma (NSG) mice. Once the tumor was established, the resulting tumor was reimplanted into additional mice for the analyses. The following antibodies were used to characterize resulting tumors: ER (clone EP1, IR 084, Dako), GATA3 (sc-268, Santa Cruz Biotechnology), FOXA1 (sc-6553, Santa Cruz Biotechnology) and IHC was performed as described previously (14). The MDA-MB-436 cell line was purchased from ATCC (#HTB-130). KTB6 and KTB34, which are two immortalized breast epithelial cell lines with luminal breast epithelial cell gene expression patterns, have been described previously (15). Transformation of these cell lines with HRAS^G12V^ + SV40-T/t antigens or PIK3CA^H1047R^ + SV40-T/t antigens has also been described previously (14). We initially established primary tumors from these cell lines in NSG mice and established primary tumors were processed into cell lines to enrich for fully transformed cells as described previously (14). To examine cytokine/chemokine/growth factor secretion from transformed cells, cells were grown to approximately 80% confluency, spent culture media were discarded, cells were washed twice with PBS, and cultured in FBS free primary cell culture media without growth factors and additives for 24 hours. All cell lines in the lab have been subjected to cell line authentication using commercial Human 16-Marker Profile and to *Mycoplasma* testing (IDEXX Bioanalytics) on December 14, 2023.

### Animal Models and Behavioral Tests

To generate PDX breast cancer models, freshly collected or cryopreserved tumors were implanted into mammary fat pads of NSG mice. All mice received 60 days slow release estradiol pellet (Innovative Research of America SE-121). Tumor sizes were monitored periodically. Once the tumors were established, tumors were harvested, divided into multiple portions and reimplanted into new mice to establish PDX lines. Control mice with estradiol pellets but no tumors were maintained for the same duration. To generate breast cancer model from human breast epithelial cells transformed with mutant oncogenes, 1 × 10^6^ freshly harvested transformed cells with 50% Matrigel (in 100 µL volume, Corning 354234) were injected into mammary fat pads of NSG mice. To establish the cell line-derived xenograft model, 5 × 10^5^ MDA-MB-436 cells were implanted into the mammary fat pad of 6–8 weeks old female nude mice. Tumor growth rate was recorded periodically. Immediately prior to termination, the animals were tested for body composition, including body lean mass, fat content, free water, and body total water (7). Grip strength and rotarod performance were measured before the termination of mice (16). Following euthanasia, tumors, blood, and skeletal muscles from hind limbs were harvested and stored at −80°C for molecular analyses. To adhere to 3Rs of animal studies, animals in MDA-MB-436 model are the same as our previously published study in which we described tumor growth patterns and response to CBL0137 treatment (17). The NIH regulations concerning the use and care of experimental animals were followed while conducting animal studies and the Indiana University School of Medicine Animal Care and Use Committee approved this study.

### Human Cytokine Array

The cytokine array was conducted using cell culture supernatants of transformed cells with the Proteome Profiler Human XL Cytokine Array Kit (R&D Systems, #ARY022B), following manufacturer's instructions. This array detects 105 cytokines/chemokines/growth factors simultaneously. Spot pixel density was quantified by NIH Image J 1.X protein array analyzer software. Values from the negative reference spots (background) were deducted from the spot pixel density values of the positive datapoints. The resulting values were then normalized as a percentage to the average pixel density values from the six built-in positive reference points. The cytokine array experiment was performed twice, and the duplicate spotting of each parameter allowed for statistical analyses. Only values higher than 5% of controls were considered for the analysis.

### Plasma Cytokine Profiling

Levels of circulating cytokines and chemokines were measured as described previously (9, 16). The MILLIPLEX MAP Mouse Cytokine/Chemokine Magnetic Bead Panel kit (MCYTMAG-70K-PX32; Millipore) was used to measure 32 cytokines/chemokines from 100 µL of plasma. Levels of TGFβ1, TGFβ2, and TGFβ3 were measured separately. Samples with no detectable values were given a score of zero for the analysis.

### Measurements of Skeletal Muscle-specific Force

Intact extensor digitorum longus (EDL) muscles from mice were used to test skeletal muscle contraction force as described previously (9). Briefly, freshly isolated EDL muscles were attached to an isometric force transducer (model 407A; Aurora Scientific) between platinum stimulating electrodes in a glass chamber containing modified Tyrode buffer. A force-frequency relationship was determined by measuring muscle contraction force at a range of frequencies (1–200 Hz; model 701C; Aurora Scientific) to obtain a maximum tetanic force plateau. Dynamic Muscle Control software and Dynamic Muscle Analysis software (dmc version 5.300, dma version 5.010; Aurora Scientific) were used for stimulation and analysis. The specific force at various stimulating points from individual EDL muscle were analyzed and averaged by group.

### Total RNA Extraction

Total RNA isolation from skeletal muscles and plasma has been described previously (7, 18). Briefly, approximately 30 mg tibialis anterior muscle were disrupted and homogenized in 700 µL QIAzol lysis reagent (Qiagen 217004) with TissueLyser, then incubated at room temperature for 5 minutes, followed by addition of 140 µL chloroform for 2 minutes, and centrifugation for 15 minutes at 12,000 × *g* at 4°C. The upper clear aqueous phase was transferred to a new tube. 1.5 volumes of 100% ethanol was added, then washed with RWT and RPE buffer with an RNA collection column. Total RNAs were eluted with RNAase free water into a new tube. To extract total RNA from plasma, 200 µL of plasma samples were aliquoted to new tubes, 700 µL QIAzol lysis reagent was added, mixed, and incubated for 5 minutes. The remaining procedures were done as described for total RNA extraction from muscles.

### qRT-PCR

The TaqMan miRNA Reverse Transcription Kit (Applied Biosystems, #4366597) was used to synthesize cDNAs with 5 µL of miRNAs (20–200 ng/µL) extracted from plasma and mouse muscle tissue. Bio-RAD iScript cDNA synthesis kit (#170-8891) was used to reverse transcribe total RNAs (50–200 ng/µL) extracted from mouse muscle to synthesize cDNAs in a 20 µL reaction. qRT-PCR was performed using TaqMan universal PCR master mix (Applied Biosystems, #4324018) and specific primers as described in our previous study (7, 9). *U6* small RNA (TaqMan, 001973), was used as normalization controls for measurement of miR-486-5p (TaqMan, #001278). *Gapdh* (TaqMan, #Mm03302249_g1) was used as a normalization control for *Pax7* (TaqMan, #Mm01354484_m1), *Myod* (TaqMan, #Mm00440387_m1), *Myh1* (TaqMan, Mm01731290_g1), *p53* (TaqMan, #Mm00440387_m1), and *Pdk4* (TaqMan, #Mm01166879_m1) mRNA measurements in skeletal muscles.

### Western Blotting

Approximately 30 mg tibialis anterior muscle were lysed in RIPA buffer containing protease/phosphatase inhibitors (Cell Signaling Technology, #5872S) with TissueLyser. A total of 30 µg of protein were used for Western blotting. Primary antibodies against p-AKT (S473, Cell Signaling Technology, #4060, rabbit), AKT (Cell Signaling Technology, #4691, rabbit), PTEN (Cell Signaling Technology, #9188, rabbit), pp38 (Cell Signaling Technology, #4511S, rabbit), and p38 (Cell Signaling Technology, #8690S, rabbit) were used for Western blots. Quantification of Western blotting was performed using NIH Image J software. For unknown reasons, only in cases of skeletal muscle extracts, we were not able to detect clear signals for commonly used loading control markers such as β-actin upon reprobing the blots. Therefore, Ponceau S staining of the blots was performed to ensure relatively equal loading of proteins in each lanes. Because phosphorylated proteins are sometimes not equally recognized by antibodies that detect total proteins, we did not use total protein corresponding to phosphoprotein for normalization of phosphoproteins. Therefore, quantitative levels of phosphoprotein and total proteins of protein-of-interest are presented separately.

### Statistical Analysis

Data were analyzed using the GraphPad Prism software 10.1.0. The two-tailed unpaired Student *t* test or one-way ANOVA with Tukey multiple comparisons post hoc test was used to compare groups as appropriate. A *P* value <0.05 was considered statistically significant.

### Data Availability

No high throughput data are presented in this article. Unprocessed Western blots and other raw data used to generate graphs have been submitted along with the article as Supplementary Data S1 and S2. Figure legends have details of blots which we reprobed for additional markers. For reagents, please contact the corresponding author.

## Results

### The Effects of Breast Cancer Subtypes on Skeletal Muscle Function

**FIGURE 1:**
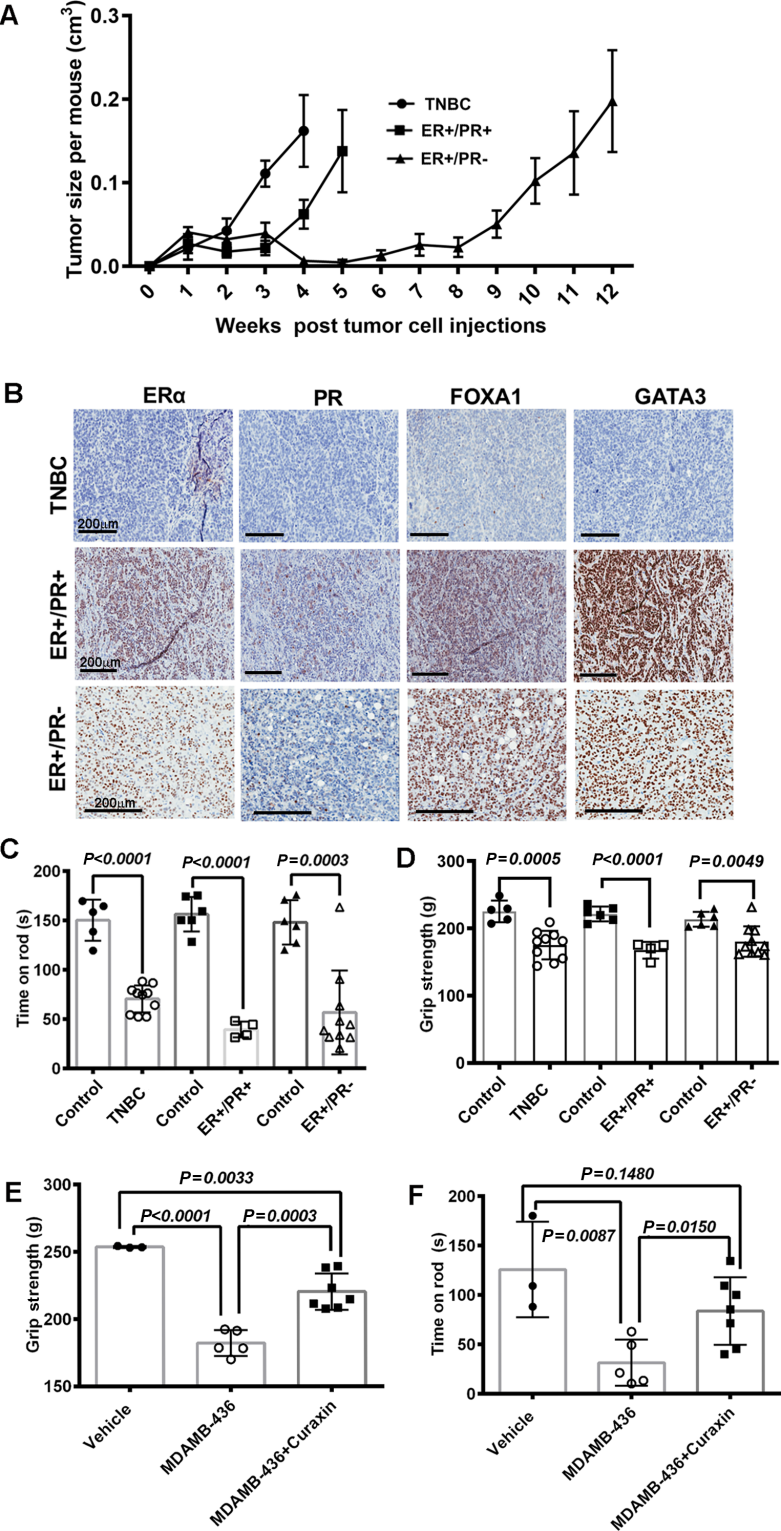
Tumor subtypes differentially affect skeletal muscle function in mice with PDXs. **A,** Growth rate of PDXs in NSG mice. **B,** Histology of PDXs confirming their molecular subtype. **C,** Impaired rotarod performance of mice with PDXs across all tumor subtypes. **D,** Differential impact of tumors by subtype on grip strength of mice with PDXs. Mice with TNBC tumors had severe loss of grip strength. **E,** Loss of grip strength was improved by Curaxin (CBL0137) treatment in nude mice with tumors from MDA-MB-436 cells. **F,** Impaired rotarod performance was ameliorated by Curaxin (CBL0137) in nude mice with tumors from MDA-MB-436 cells.

To understand whether breast cancer subtypes differentially affect skeletal muscle dysfunction, we selected three PDXs representing three major subtypes of breast cancer: TNBC, luminal A (ER+/PR+), and luminal B (ER+/PR−). Growth patterns of these PDXs are shown in Fig. 1A. As expected, TNBC-derived PDX grew faster than luminal A or luminal B tumor-derived PDXs. Slower growth rate of ER+/PR− tumor compared with ER+/PR+ tumor is likely due to generation of these tumors using tumor cells suspended in pleural effusion and as such lack the primary tumor microenvironment. IHC of these PDXs for ER, PR, FOXA1, and GATA3 to confirm their molecular identities is shown in Fig. 1B. The molecular identities of the TNBC PDXs were determined in tumors derived from reimplanted PDXs whereas the primary PDXs were assessed in cases of ER+/PR+ and ER+/PR− tumors.

To evaluate the impact of these tumor subtypes on skeletal muscle, we tested skeletal muscle function and harvested tissues when the tumors reached similar sizes from the three groups (∼0.2 cm^3^). In all three PDX models, rotarod performance was reduced suggesting that all breast tumor subtypes impact skeletal muscle performance (Fig. 1C). All three PDX models reduced grip strength (Fig. 1D). To further extend these observations, we established tumors in nude mice with MDA-MB-436 cell line, a TNBC cell line (19). Tumor-bearing mice demonstrated reduction of grip strength (Fig. 1E) and the impairment of rotarod performance (Fig. 1F). We had previously demonstrated that MDA-MB-436–derived tumors are highly sensitive to the FACT complex inhibitor Curaxin (CBL0137; ref. 17). In addition to a decrease in tumor volume (0.12 cm^3^ in treated compared with 1.38 cm^3^ in untreated controls at the time of sacrifice), treatment with CBL0137 resulted in a partial, yet substantial restoration of muscle function (Fig. 1E and F). These results suggest that the level of skeletal muscle dysfunction is proportional tumor burden and is reversible with effective treatment.

### Tumor Subtype-specific Effects on Skeletal Muscle

**FIGURE 2:**
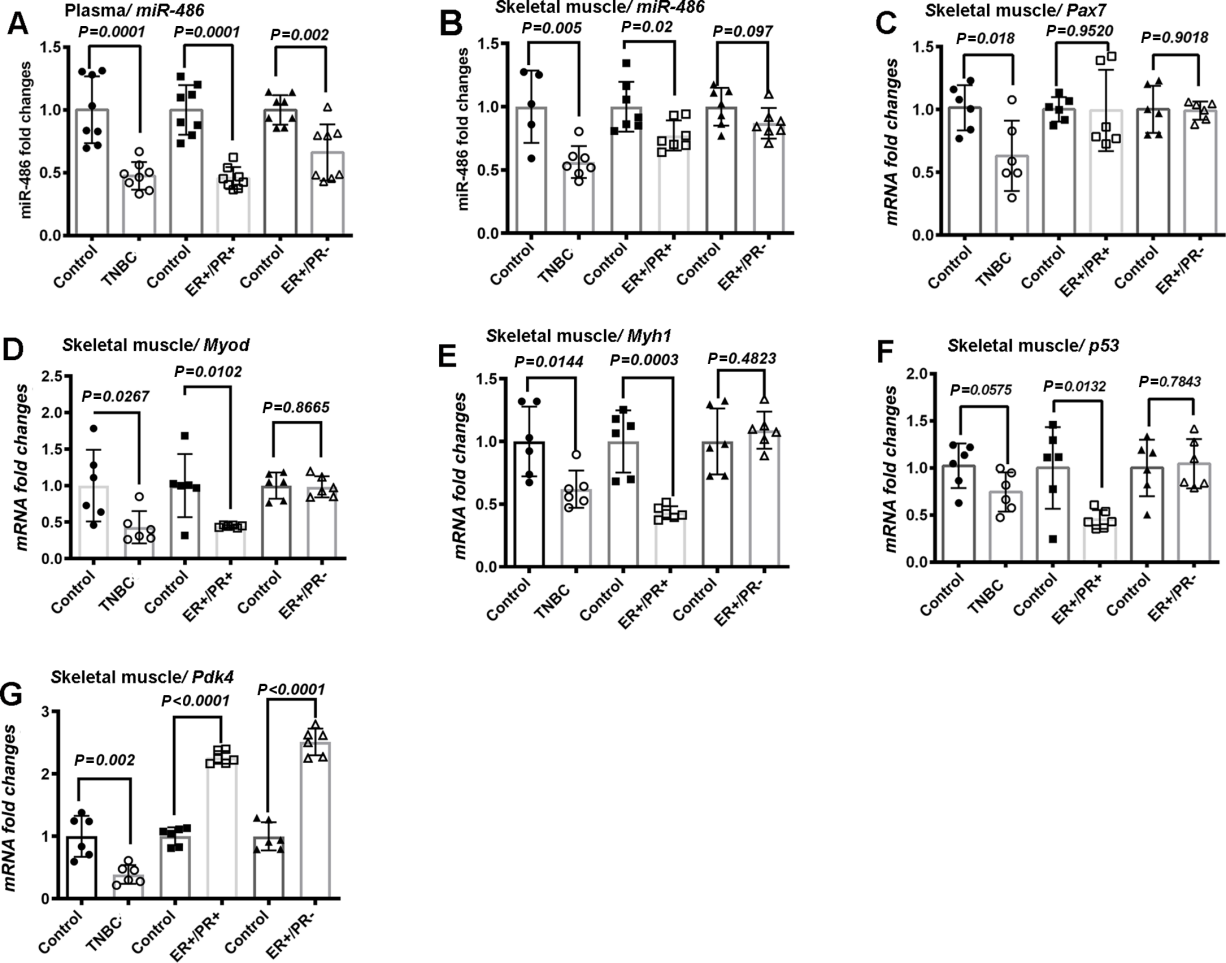
The effects of PDXs on plasma *miR-486* and functional markers in skeletal muscle. **A,** A significant decline in circulating *miR-486* levels was observed across all PDX subtypes. **B,** Differential effects of PDX subtypes on *miR-486* in skeletal muscles of mice. **C,** Differential impact of PDXs on skeletal muscle *Pax7*. **D,** Differential impact of PDXs on skeletal muscle *MyoD*. **E,** The effect of PDXs on skeletal muscle *Myh1*. **F,** The effect of PDXs on skeletal muscle *p53*. **G,** ER+/PR+ and ER+/PR− tumor-derived PDXs increased skeletal muscle *Pdk4*, compared with TNBC PDXs.

Consistent with functional skeletal muscle defects noted above, we found that circulating *miR-486*, a crucial player in myogenesis and in the maintenance of skeletal muscle integrity (20, 21), was lower in PDX-bearing mice compared with control mice (Fig. 2A). Skeletal muscle *miR-486* was significantly lower in mice with TNBC and ER+/PR+ PDXs, while no significant difference was observed in ER+/PR− PDXs (Fig 2B). Next, we examined expression changes in genes that are associated with myogenesis and muscle function. mRNA levels of *Pax7*, *Myod*, *Myh1*, *p53*, and *Pdk4* were measured for the following reasons. Both PAX7 and MyoD are essential regulators of homeostasis, proliferation, and differentiation of skeletal muscle stem cells (22, 23). MYH1 is mainly expressed in fast contraction muscle fibers (24) and is a marker of myogenic differentiation (25). p53 regulates mitochondrial function of skeletal muscles (26–28) and we had previously demonstrated its reduced expression in skeletal muscle of transgenic models of breast cancer (9). Upregulation of PDK4 in skeletal muscle has previously been reported in models of cachexia (29). We observed that *Pax7* was significantly downregulated in skeletal muscle of mice with TNBCs, but not with ER+/PR+ and ER+/PR− tumors (Fig. 2C). Downregulation of *Myod*, *Myh1*, and *p53* mRNA levels was observed in TNBC and ER+/PR+ tumor-bearing mice, compared with ER+/PR− PDXs which showed no significant difference, relative to control (Fig. 2D–F). Surprisingly, we observed downregulation of *Pdk4* in mice with TNBCs, but its upregulation in skeletal muscle of ER+/PR+ and ER+/PR− tumor-bearing mice (Fig. 2G). These results suggest that each tumor subtype can cause distinct changes in skeletal muscle.

**FIGURE 3:**
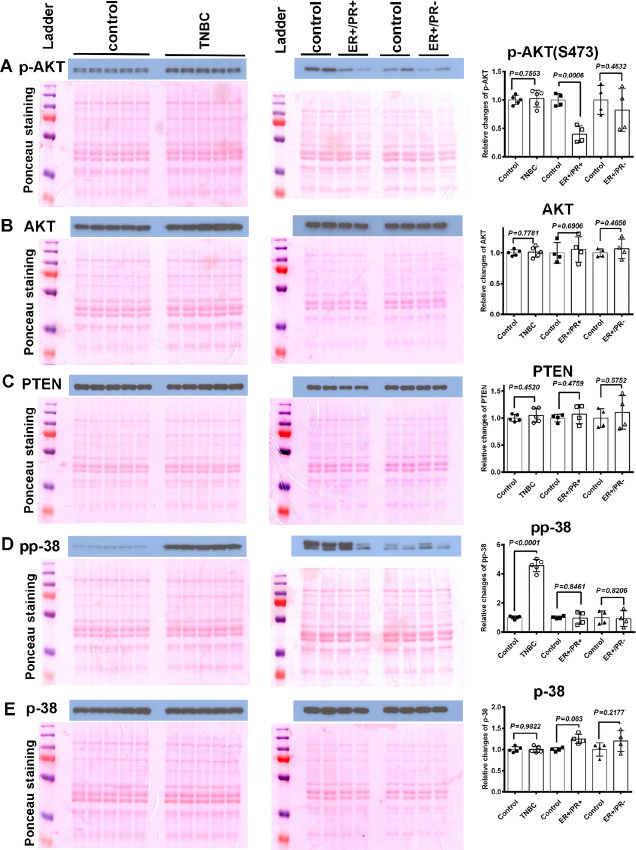
Impact of PDXs on activation status of select signaling molecules in skeletal muscle. **A,** Tumors from ER+/PR+ tumor cells reduced AKT phosphorylation in skeletal muscle. **B,** AKT protein levels in skeletal muscles were not affected by tumors. **C,** PTEN protein levels in skeletal muscles were not changed by tumors, compared with the control group. **D,** TNBC-derived PDX upregulated phospho-p38 in skeletal muscle. **E,** Tumors did not affect total p-38 protein levels in skeletal muscle. Quantification of western blotting was performed using NIH ImageJ software 1.X.

To further explore tumor-specific effects on skeletal muscle, we examined the levels of previously reported mediators of cancer-induced skeletal muscle defects. Previous studies have shown that alterations in AKT signaling are involved in skeletal muscle dysfunction (16, 30). Representative Western blot data from two/three animals per group but quantitative data from 4/5 animals per group are shown in Fig. 3. We observed no change in pAKT levels in skeletal muscles of mice with TNBC PDXs but found a significant decrease of pAKT in skeletal muscle of mice with ER+/PR+ PDXs. Skeletal muscle of mice with ER+/PR− PDXs showed a downward trend but differences did not reach statistical significance (Fig. 3A). We did not observe significant changes of total AKT and its upstream regulator PTEN protein levels in skeletal muscles of mice in all three groups (Fig. 3B and C). These data suggest that TNBC PDXs induce alternative pathways that are distinct from AKT signaling to affect skeletal muscle function. Indeed, skeletal muscle of mice bearing TNBC PDXs displayed higher levels of phospho-p38 (Fig. 3D), which has previously been shown to be upregulated in cachectic muscles (31, 32). Changes of total p38 level were not observed in skeletal muscles of mice in all three groups (Fig. 3E). These results indicate that breast cancer subtypes differentially affect signaling pathways in skeletal muscles.

### Breast Epithelial Cells Transformed with Specific Oncogenes Differentially Impact Skeletal Muscle Function

**FIGURE 4:**
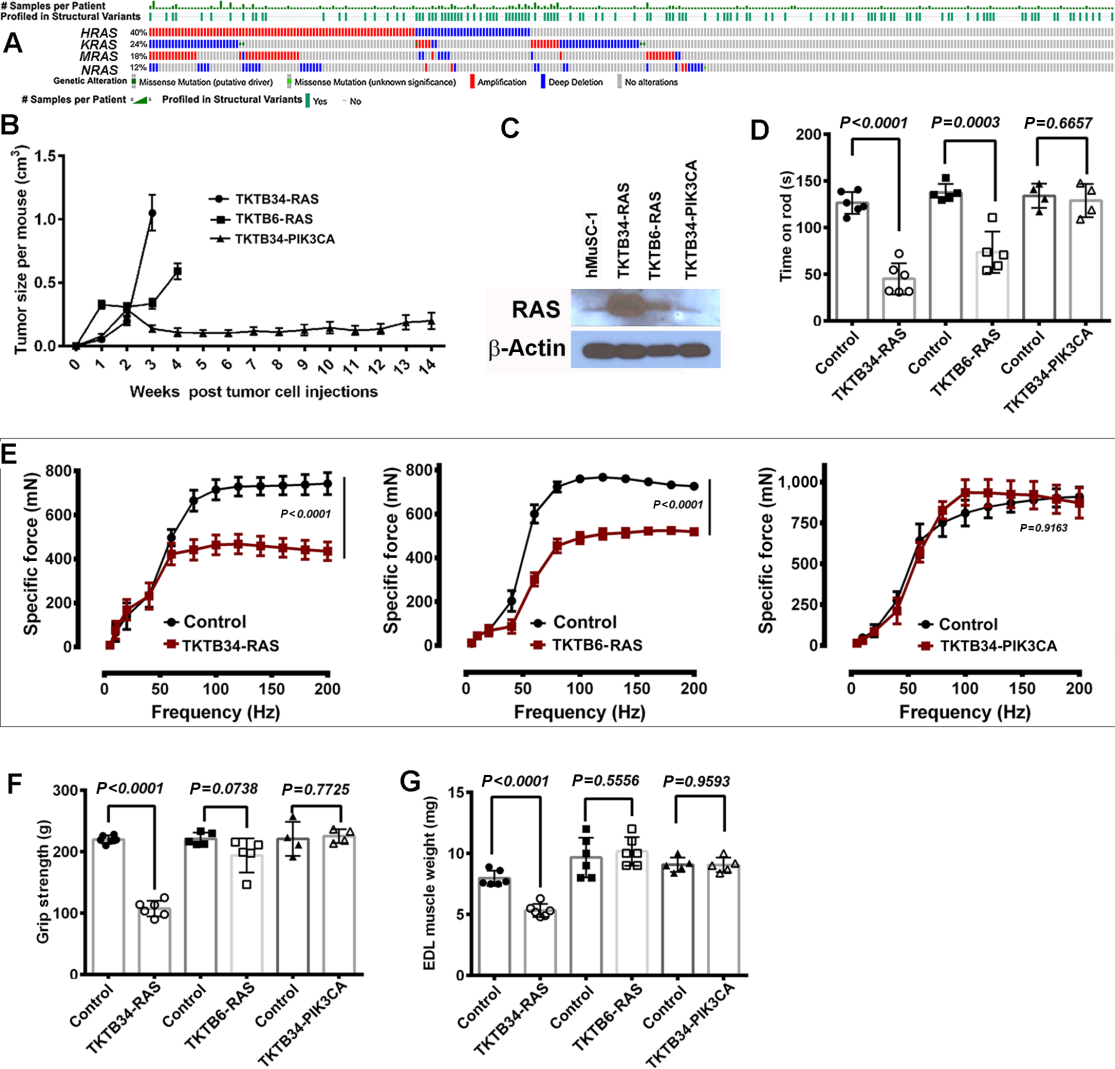
Tumors derived from breast epithelial cells transformed with HRAS^G12V^ oncogene cause significant skeletal muscle defects. **A,** Metastatic breast cancers show significant genomic aberrations in four *RAS* proto-oncogenes. **B,** Growth rate of tumors derived from three cell lines—TKTB34-RAS, TKTB6-RAS, and TKTB34-PIK3CA. A total of 1 × 10^6^ cells were injected to the mammary fat pad. **C,** RAS protein levels in transformed cell lines. **D,** Impaired rotarod performance were observed in mice bearing tumors from RAS transformed cells. **E,** Mutant RAS oncogene-derived tumors but not mutant PIK3CA-derived tumors caused reduction in skeletal muscle contraction force. **F,** Tumors derived from TKTB34-RAS, which expressed higher levels of HRAS^G12V^ than TKTB6-RAS, caused severe loss of grip strength. **G,** Tumors derived from TKTB34-RAS transformed cells caused reduction in EDL muscle weight.

Although PDXs recapitulate most of the characteristics of human breast tumors, it is difficult to attribute PDX-induced changes in skeletal muscle to specific genomic aberrations in tumors, as these PDXs typically have multiple genomic aberrations including passenger mutations. Therefore, a tumor model system generated using defined oncogenes is needed to identify skeletal muscle changes attributable to specific genomic aberrations. Toward this goal, we generated three different transformed cell lines using breast tissues from healthy donors (14). Immortalized cell lines generated from breast tissues of two clinically healthy European ancestry donors were used (KTB6 and KTB34; ref. 15). Transformation of these cell lines with HRAS^G12V^ or PIK3CA^H1047R^ along with SV40-T/t antigens has been described previously (14). We selected HRAS oncogene because a significant fraction of metastatic breast cancers demonstrate *HRAS* amplification based on data extracted from metastatic breast cancer sequencing project in cBioportal (Fig. 4A; ref. 13). In another study focused on metastatic breast cancer, genomic aberrations involving *HRAS, KRAS, MRAS*, and *NRAS* was observed in 14% of cases (33). Furthermore, genomic aberrations involving the *NF1* gene, which leads to higher RAS activity (34), is observed in 15% of metastatic breast cancers. Tumors derived from all three cell lines were adenocarcinomas (14) and showed different growth rate (Fig. 4B). Note that TKTB6-HRAS^G12V^ + SV40-T/t (labeled TKTB6-RAS hereafter) expressed much lower levels of mutant RAS oncogene than TKTB34-HRAS^G12V^ + SV40-T/t antigens (labeled TKTB34-RAS hereafter; Fig. 4C) and that only TKTB34-RAS–derived tumors metastasized to lungs (14). Tumors derived from TKTB34-PIK3CA^H1047R^ + SV40T/t (labeled TKTB34-PIK3CA hereafter) grew slowly and did not generate overt metastasis (Fig. 4B; ref. 14). Also note that TKTB34-RAS and TKTB34-PIK3CA tumor cell lines have the same genetic background as they are derived from cells of the same donor and any differences in their properties are largely due to differences in oncogenic aberrations. Mice with RAS-derived tumors but not with mutant PIK3CA-derived tumors demonstrated reduced rotarod performance (Fig. 4D) and muscle contraction force (Fig. 4E). Potentially owing to higher mutant HRAS expression and lung metastasis, mice with TKTB34-RAS cell-derived tumors but not others demonstrated reduced grip strength (Fig. 4F) and lower EDL weight (Fig 4G).

### Molecular Changes in Skeletal Muscle of Mice with Transformed Cell-derived Tumors

**FIGURE 5:**
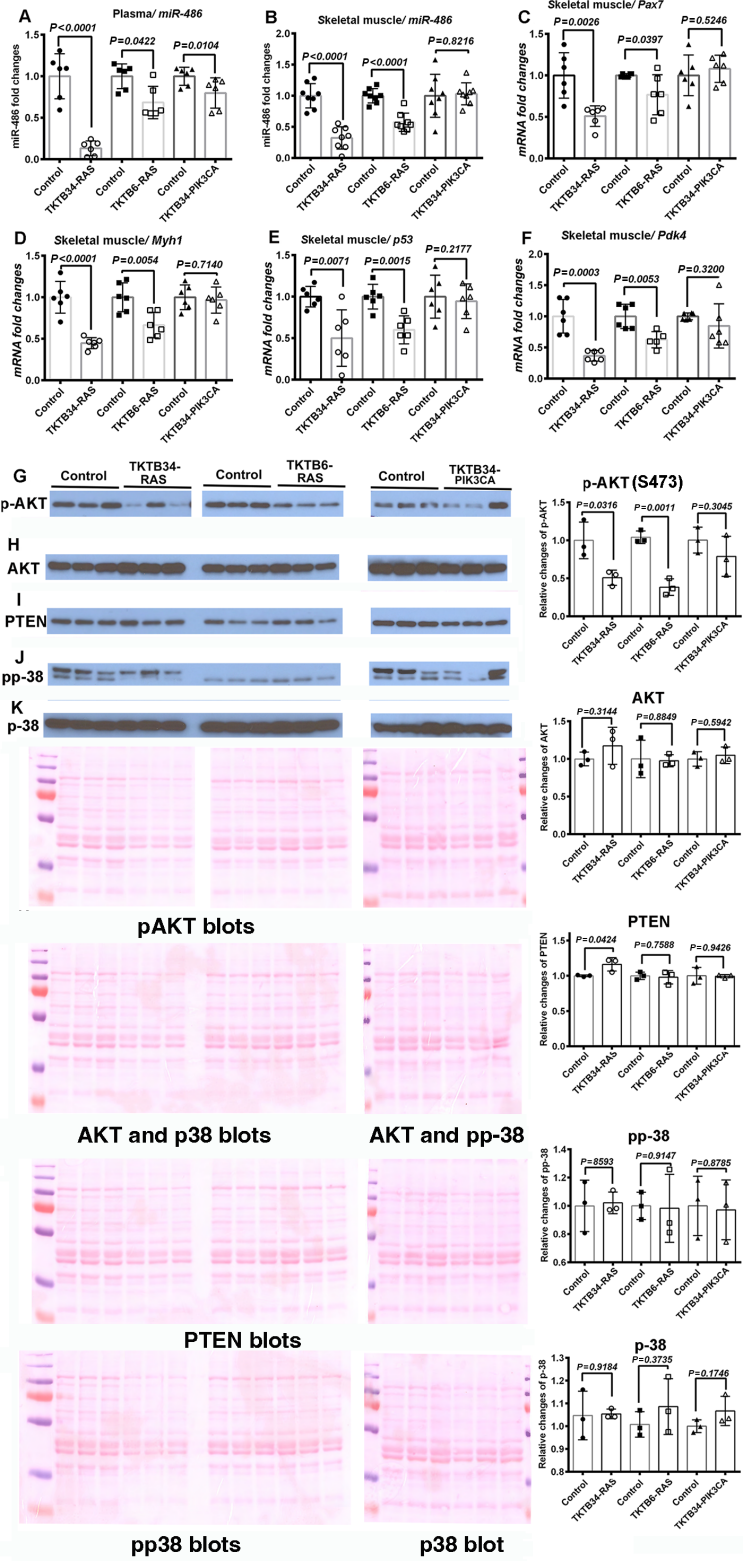
Molecular changes in plasma and skeletal muscles of mice bearing tumors initiated from transformed cell lines. **A,** Plasma *miR-486* levels. **B,** Skeletal muscle *miR-486* levels. **C,** Skeletal muscle *Pax7* mRNA levels. **D,** Skeletal muscle *Myh1* mRNA levels. **E,** Skeletal muscle *p53* mRNA levels. **F,** Skeletal muscle *Pdk4* mRNA levels. **G,** Skeletal muscle phosphorylated AKT levels. **H,** Total AKT levels. **I,** PTEN levels. **J,** Phosphorylated p-38 levels. **K,** Total p-38 levels. Quantification of Western blotting was performed using NIH ImageJ software 1.X. Note that in case of TKTB34-PIK3CA, the same blot was probed for AKT and pp38 and, therefore, one Ponceau S blot is shown. Similarly, the same TKTB34-RAS and TKTB6-RAS blot was probed with AKT and p-38 antibodies and one Ponceau S blot is shown.

As observed in PDX models, all tumor-bearing mice demonstrated reduced levels of circulating *miR-486* (Fig. 5A). However, substantial molecular changes in skeletal muscles were observed in only HRAS-derived tumors. For example, decline in skeletal muscle *miR-486* (Fig. 5B), *Pax7* (Fig. 5C), *Myh1* (Fig. 5D), *p53* (Fig. 5E), and *Pdk4* (Fig. 5F) was observed in mice with TKTB34-RAS and TKTB6-RAS cell-derived tumors but not in TKTB34-PIK3CA cell-derived tumors. Decline in skeletal muscle pAKT was found to be statistically significant only in mice with TKTB34-RAS and TKTB6-RAS cell-derived tumors (Fig. 5G). Significant alterations of AKT, PTEN, pp-38, and p38 were not observed in all three groups of mice (Fig. 5H–K).

### Transformed Cells with Specific Oncogenes Secrete Unique Cytokines/Chemokines/Growth Factors

**FIGURE 6:**
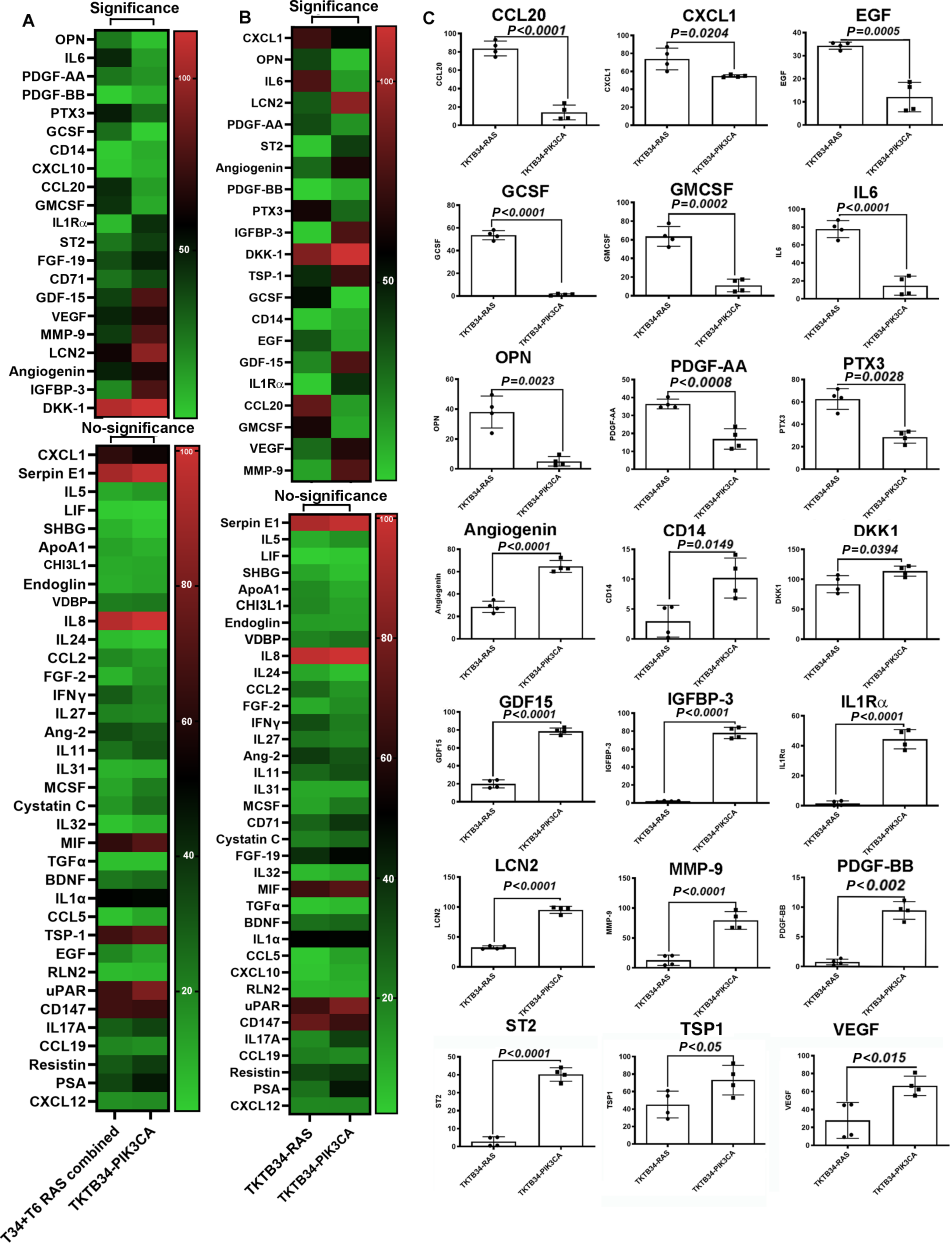
Cytokines/chemokines/growth factors secreted from cells transformed with mutant HRAS or PIK3CA. The assay was done twice in duplicate and intensity of the signals was normalized to internal controls in the array. Those with signals >5% of internal controls were considered for statistical analysis. **A,** Heat map of cytokines/chemokines/growth factors in array that compared data from TKTB34-RAS+TKTB6-RAS with TKTB34-PIK3CA. Statistically significant differences (*P* < 0.05) are shown on top. **B,** Same as in A except that comparison was limited to TKTB34-RAS with TKTB34-PIK3CA. **C,** Select cytokines/chemokines/growth factors differentially released by TKTB34-RAS and TKTB34-PIK3CA cells.

Cancers with oncogenic *RAS* mutations have been shown to modulate the immune system through secretion of specific cytokines and chemokines (35). Cytokines play important roles in cancer-associated skeletal muscle defects (21, 36). Therefore, we examined the secreted cytokines/chemokines/growth factors in culture supernatants from three groups of transformed cells using a cytokine/chemokine/growth factor array. To allow statistical analyses, the study was repeated to obtain four datapoints (each array has duplicate spots). Data were analyzed by comparing TKTB34-RAS + TKTB6-RAS with TKTB34-PIK3CA (Fig. 6A) or TKTB34-RAS with TKTB34-PIK3CA (Fig. 6B). The second analysis is necessary because of recent observations of genetic ancestry-dependent and interindividual variability in cytokine production (37, 38). Release of 17 cytokines/chemokines/growth factors was significantly different between RAS transformed and PIK3CA transformed cells in both the analyses. Several of the factors differentially expressed between TKTB34-RAS and TKTB34-PIK3CA are shown in Fig. 6C. Supplementary Figure S1 provides data on expression levels of other secreted factors not included in Fig. 6C. RAS-transformed cell enriched factors include CCL-20, CXCL1, EGF, GCSF, GMCSF, IL6, OPN, PDGF-AA, and PTX3. Those enriched in PIK3CA transformed cells include Angiogenin, CD14, DKK1, GDF15, IGFBP-3, IL1Rα, LCN2, MMP-9, PDGF-BB, ST2, TSP1, and VEGF.

### Circulating Cytokines/Chemokine Levels in Mice with Different Tumor Types

**FIGURE 7:**
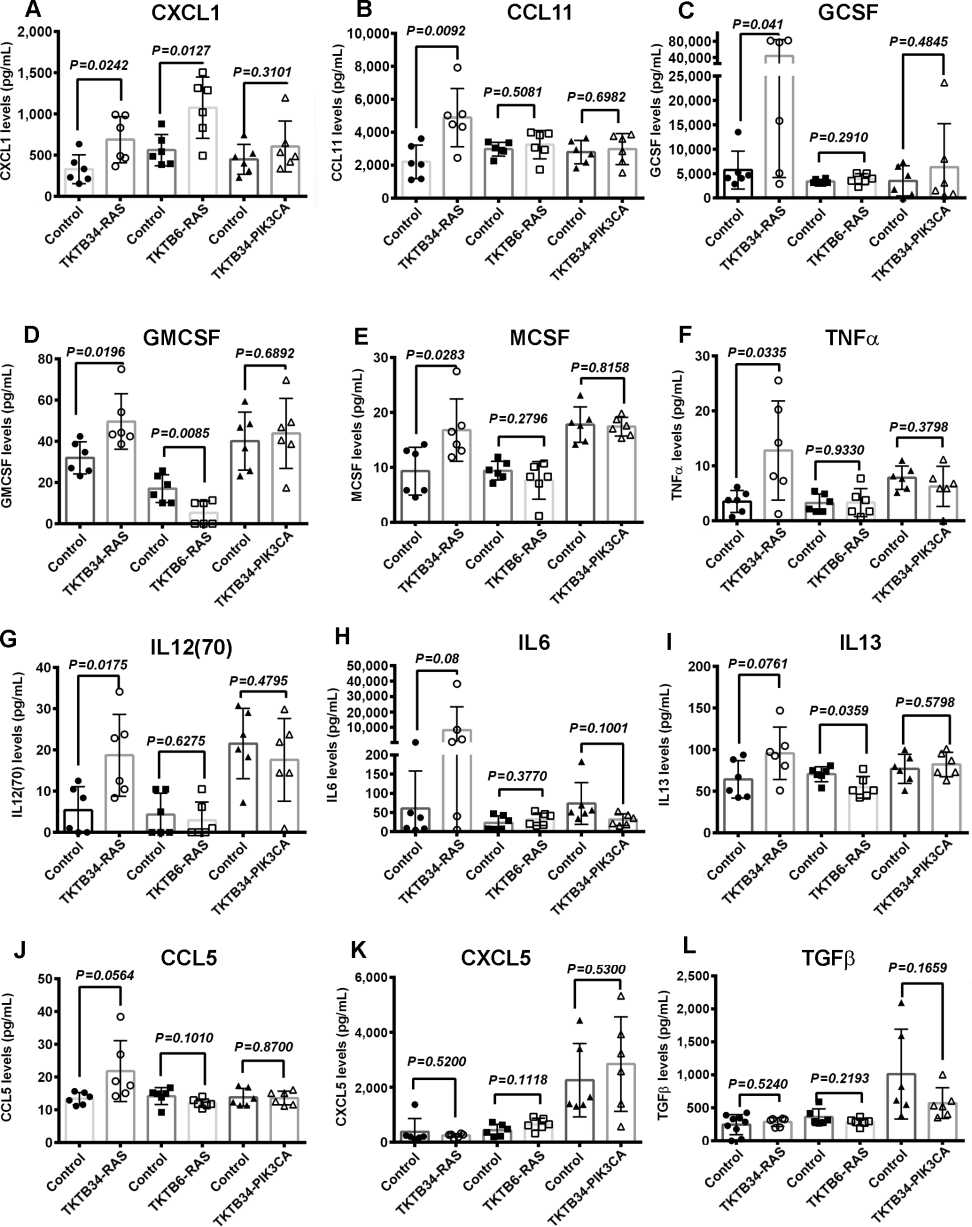
Circulating cytokine profile in plasma of control and tumor-bearing NSG mice. **A,** Increased circulating CXCL1 in mice bearing tumors from TKTB34-RAS and TKTB6-RAS cell lines compared with control mice. **B–J,** Elevated circulating cytokine levels of CCL11, GCSF, GMCSF, MCSF, TNFα, and IL12(70) in mice bearing tumors from TKTB34-RAS cell line. Differences in circulating IL6, IL13, and CCL5 did not reach statistical significance. **K** and **L,** Circulating CXCL5 and TGFβ were not affected in tumor-bearing mice.

To determine whether cancer causes systemic changes in cytokine/chemokine levels, which can consequently affect skeletal muscle function, we examined 35 circulating cytokines/chemokines from the plasma of NSG mice with/without tumors from transformed cells. Elevated levels of CXCL1 were observed in TKTB34-RAS and TKTB6-RAS tumor-bearing mice compared with control groups without tumors (Fig. 7A). CXCL1 levels were not elevated in mice with TKTB34-PIK3CA–derived tumors (Fig. 7A). We found that circulating levels of CCL11, GCSF, GMCSF, MCSF, TNFα, and IL12 (p70) were higher in TKTB34-RAS tumor-bearing mice compared with control group without tumors (Fig. 7B–J). Interestingly, tumors did not affect the circulating levels of CXCL5 and TGFβ among all three groups, compared with control groups without tumors (Fig. 7K and L). TGFβ is often considered as a driver of metastasis-induced skeletal muscle defects (39) but does not appear to be a major contributor to RAS-induced skeletal muscle defects.

## Discussion

Multiple factors contribute to cancer-associated muscle dysfunction, including tumor types, cancer stages, sex, age, and treatment regimen (4, 40–43). The impact of tumor type varies as prevalence of muscle wasting ranges from approximately 30% or less in cancers such as breast and prostate cancer to 70% in pancreatic cancer (4). Muscle weakness often precedes muscle atrophy during cancer cachexia suggesting that skeletal muscle dysfunction occurs even without phenotypic manifestation of skeletal muscle loss (44). Recent advancements in breast cancer research have shown that PDX tumor models preserve the genetic characteristics of primary tumors over time and are better at predicting tumor development and therapeutic response (42, 45, 46). PDX models of each breast cancer subtypes closely represent the molecular profiles and patterns of drug response of human tumors (11). In the current study, we found most severe skeletal muscle defects in mice with TNBC PDXs compared with mice with PDXs representing luminal A and luminal B subtypes. The impact of tumor subtypes on skeletal muscles were further validated using transformed human tumor cells. Mice with tumors initiated from transformed tumor cells that can metastasize to lungs (TKTB34-RAS) displayed the most severe skeletal muscle defects. These results support the notion that the aggressiveness of breast cancer subtypes correlates with severity of skeletal muscle dysfunction. At the cancer-specific molecular alterations level, we found tumors generated from mutant RAS, which is often found in metastatic breast cancers, orchestrate significant skeletal muscle defects, suggesting that secretome from RAS mutant containing tumor cells cause significant systemic damage. Because RAS pathway alterations are found in 19% of cancers (12) and several of these cancers progress to terminal cachexia, a clear understanding of mutant RAS-driven skeletal muscle changes may help to reduce systemic effects and improve outcome in many cancer types.

RAS-driven secretome responsible for skeletal muscle defect is yet to be characterized. Mutant KRAS has been shown to induce the expression of variety of cytokines/chemokines including IL6, IL1, CXCL1, CXCL2, CXCL5, CXCL8, and CCL5 (35). A large number of cytokines/chemokines are involved in cancer-associated skeletal muscle dysfunction, including loss of muscle mass, muscle weakness, and changes in metabolic processes in skeletal muscle (21, 36, 47). We found high levels of CCL-20, CXCL1, EGF, GCSF, GMCSF, IL6, OPN, PDGF-AA, and PTX3 in the culture media of TKTB34-RAS, which caused severe skeletal muscle defects, compared with the TKTB34-PIK3CA tumor line. Similarly, mice bearing tumors derived from TKTB34-RAS had higher levels of several circulating cytokines/chemokines [i.e., CXCL1, CCL11, GCSF, GMCSF, MCSF, TNFα, and IL12 (p70)]. Mice with TKTB6-RAS–derived tumors, which grew relatively slower than TKTB34-RAS–derived tumor, owing to lower HRAS^G12V^ expression levels, demonstrated elevated CXCL1 levels suggesting a correlation between circulating CXCL1 and mutant RAS-induced skeletal muscle defect. CXCL1 has previously been shown to antagonize *in vivo* muscle regeneration and interfere with muscle satellite cell homeostasis (48). CXCL1 along with other cytokines secreted as a consequence of RAS activation/amplification could lead to skeletal muscle defects. It is well documented that tumor-derived GMCSF upregulates IL6 (49), while high level of IL6 accelerates skeletal muscle atrophy (50, 51) and fat loss (52). In addition, CCL11, a factor that accelerates tissue aging (53), may exacerbate such conditions. Therefore, it is not surprising that the highly aggressive subtype of breast tumors had more profound impact on skeletal muscle dysfunction.

Our current study demonstrate that genes involved in myogenesis (i.e., *Pax7* and *Myod*) and muscle function (i.e., *Myh1* and *Pdk4*), as well as *miR-486*, a regulator of myogenesis (16, 30), are uniquely dysregulated in skeletal muscles based on breast cancer subtype and mutation pattern. These findings are consistent with a previous report of differential gene expression in skeletal muscle biopsies of patients with breast cancer by subtype (54). Compared with skeletal muscle of control group, 2,410 genes were uniquely changed in HER2+ patients, 173 in patients with TNBC, 80 in ER+/PR+/HER2+ patients and only seven genes were uniquely changed in ER+/PR+ patients (54). Similarly, we found that signaling pathways in skeletal muscles were distinctly dysregulated by specific breast cancer subtypes in PDX models, along with functional defects. The p38 MAP kinase pathway was specifically activated only in skeletal muscles of mice with TNBC PDXs, while AKT pathway was downregulated only in ER+/PR+ PDXs. We recently reported a more detailed *in vitro* analysis of human skeletal muscles exposed to secretome of breast cancer cell lines representing three major subtypes of breast cancer (55). In addition, animal models of breast cancer have demonstrated that specific subtypes uniquely dysregulate molecular signaling and function of skeletal muscle (7, 9, 16). Taken together, our data suggest that breast cancer subtype determines dysregulation of specific genes and signaling pathways in skeletal muscles during disease progression. Although many changes in cytokines, miRNAs, gene expression, signaling pathways, and morphology are shared by multiple breast cancer subtypes (9, 54, 55), our data support the notion that differentially expressed cytokines/chemokines/growth factors from host immune cells in response to tumors and/or by the tumor cells by subtype may uniquely dysregulate gene expression, signaling pathways, and muscle structures that manifest as distinct functional defects in skeletal muscles. Tumor genomic data generated as a part of precision oncology need to be explored in decision making on treatment options not only for controlling tumor progression but also for reducing systemic effects of cancer.

Studies related to mutant *RAS* oncogene and cachexia in breast cancer are often met with skepticism by reviewers because of the dogma in the field that both of these are not relevant for breast cancer. Because of this skepticism, significant progress achieved in the RAS field including the development of mutant RAS-specific inhibitors (56) is not being translated clinically in breast cancer. However, recent advances clearly indicate the role of RAS signaling in metastatic breast cancers (Fig. 4) and cachexia impacting 25% of patients with breast cancer (4). We hope results presented in this study would help to overcome this skepticism at least partially and will help to move the field forward.

## Supplementary Material

Supplementary Data S1 Unprocessed western blot images and Ponceau S staining used to generate figures in the main manuscript

Supplementary Data S2 Raw values of the each cytokine measured in plasma of control and tumor bearing mice.

Supplementary Figure S1 Shows the levels of cytokines/chemokines/growth factors released by TKTB34-RAS and TKTB34-PIK3CA cells. Related to Figure 6 of the main manuscript.
