# Supplementary material for: Mutant RAS-driven Secretome Causes Skeletal Muscle Defects in Breast Cancer
Source: Cancer Res Commun. 2024 May 15;4(5):1282–95. doi: 10.1158/2767-9764.CRC-24-0045 (PMC11094532; doi:10.1158/2767-9764.CRC-24-0045)
Supplement: Supplementary Data S1 — Unprocessed western blot images and Ponceau S staining used to generate figures in the main manuscript [file crc-24-0045-s01.pdf]

Supplementary Data S1: Unprocessed western blot images and Ponceau S staining used to generate figures in the main manuscript

Raw data for Figure 3

Raw data for PDX mice of TNBC

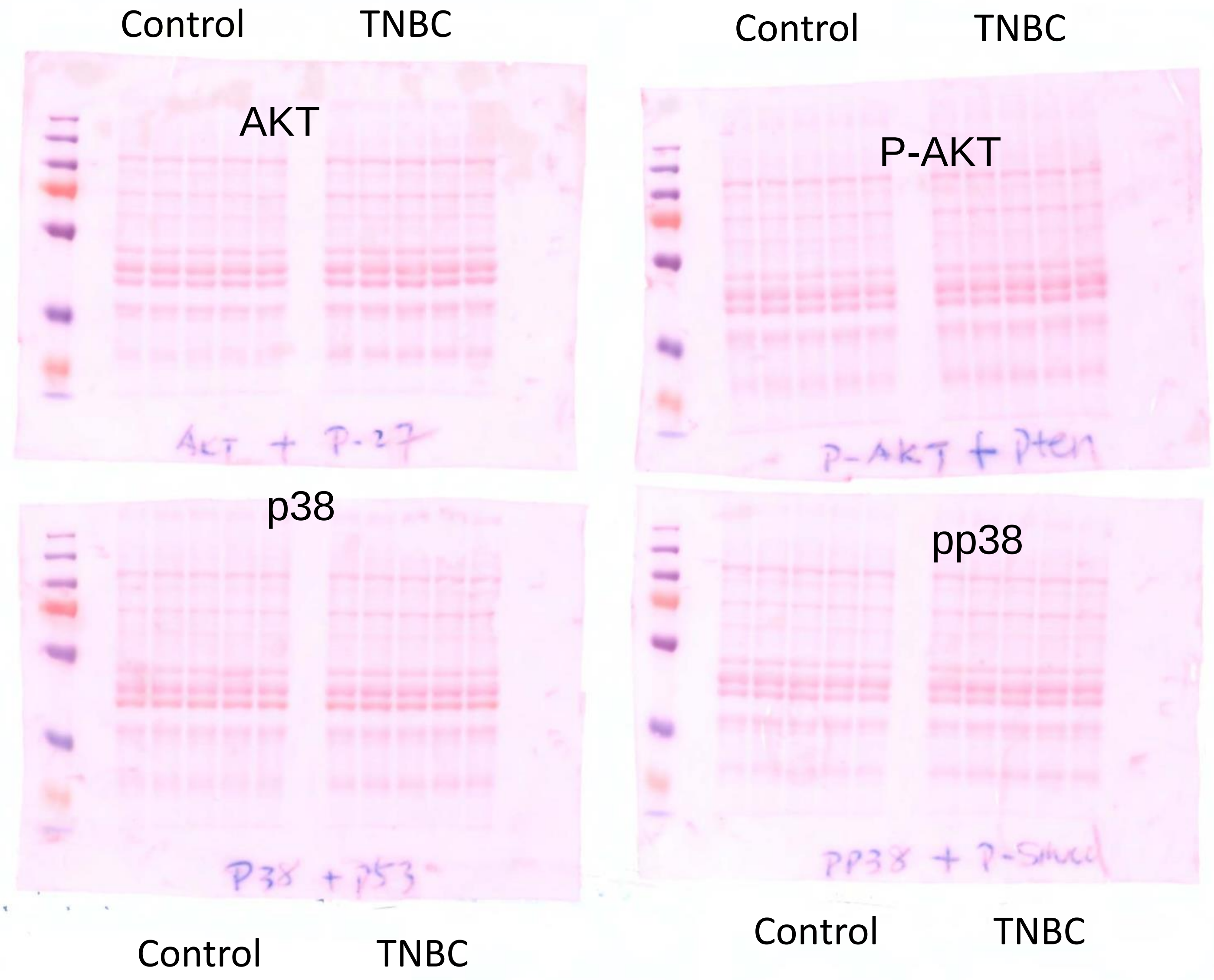
Control
TNBC
AKT
AKT + P-27
Control
TNBC
P-AKT
P-AKT + Pten
p38
P38 + p53
Control
TNBC
pp38
PP38 + P-Smad
Control
TNBC

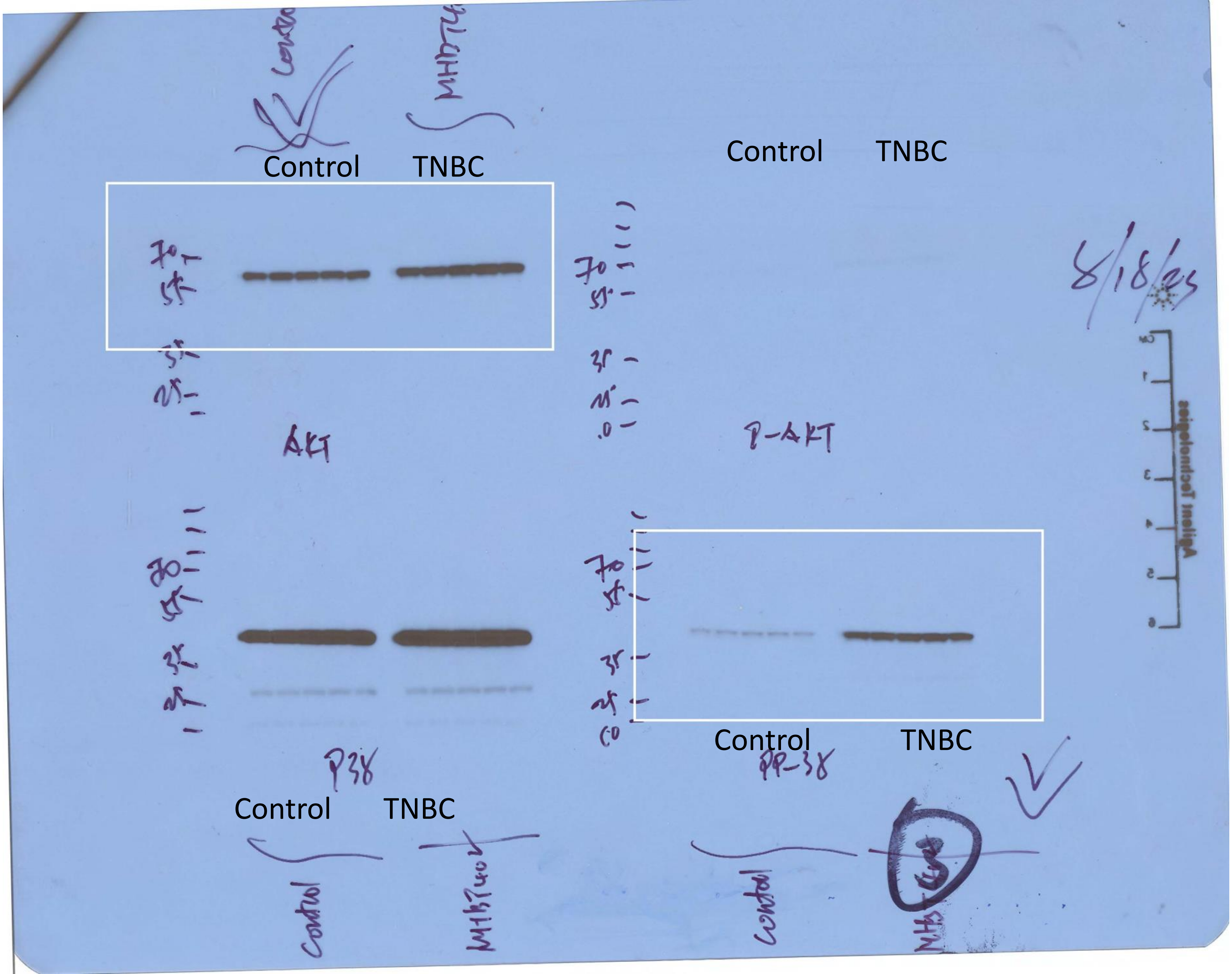
Control
TNBC
Control
TNBC
AKT
P-AKT
P38
PP-38
Control
TNBC
Control
TNBC
8/18/23

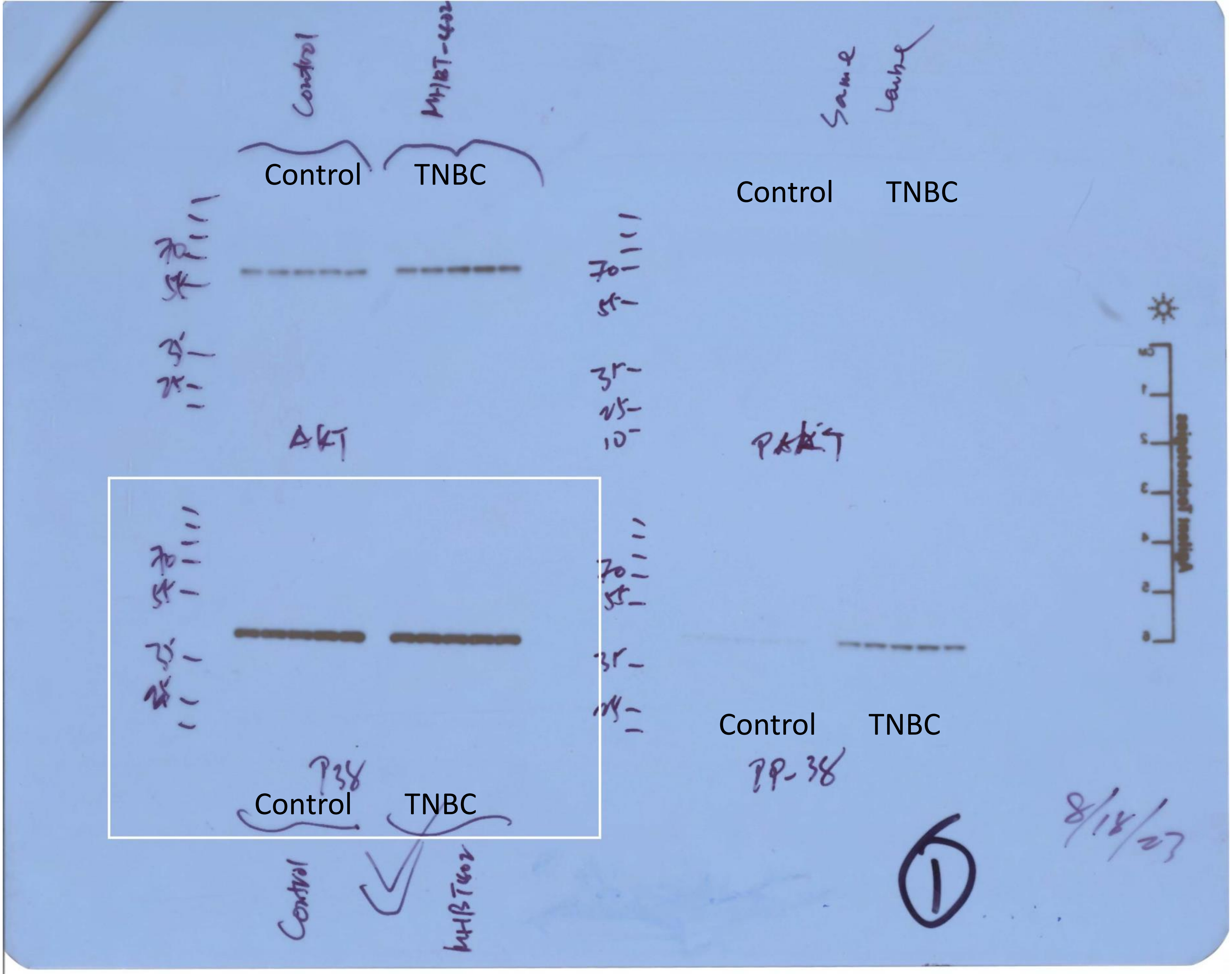
Control
TNBC
Control
TNBC
AKT
Control
TNBC
P38
Control
TNBC
8/18/23

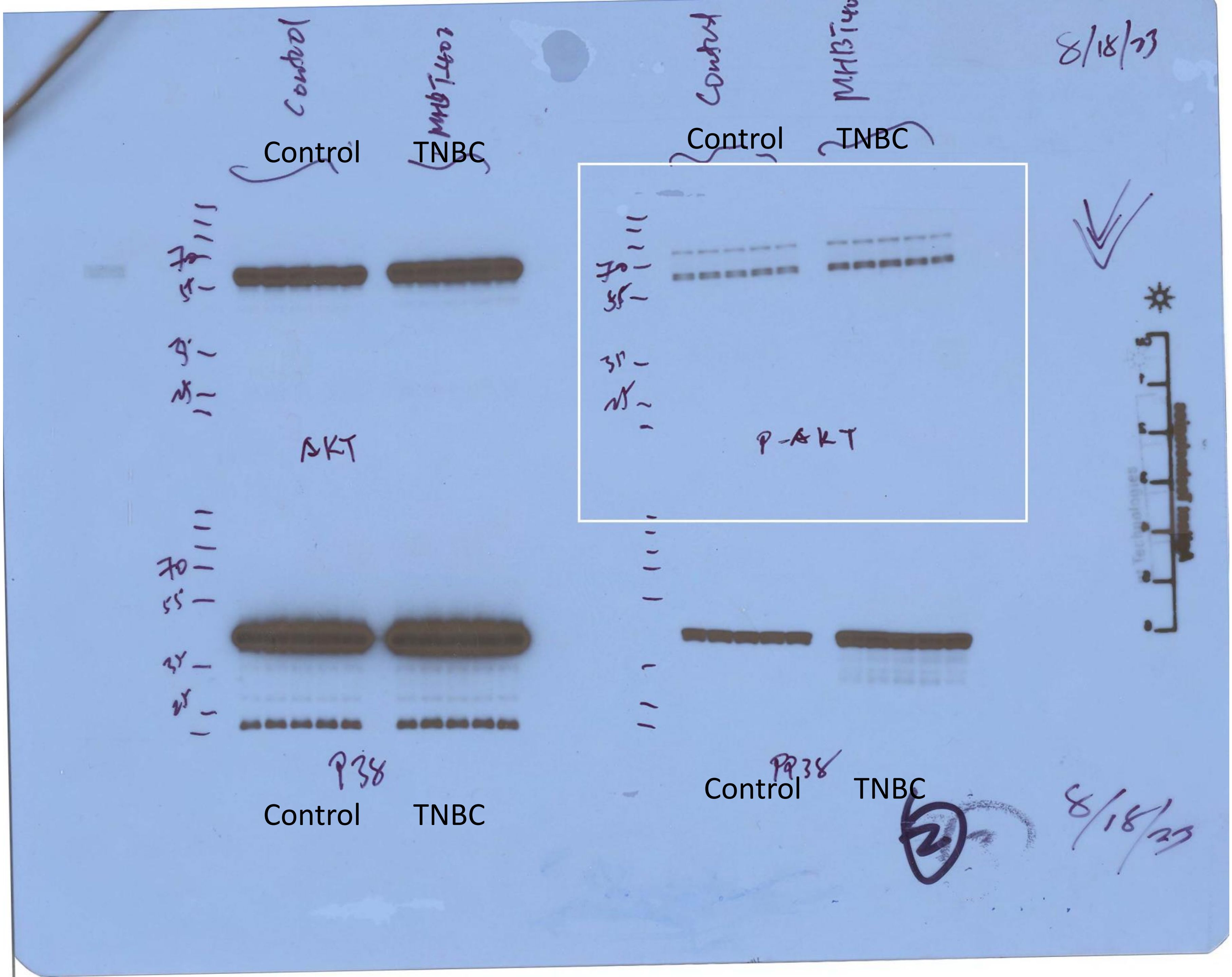
Control
TNBC
Control
TNBC
8/18/23
70
55
35
25
AKT
P-AKT
70
55
35
25
P38
Control
TNBC
P38
Control
TNBC
8/18/23

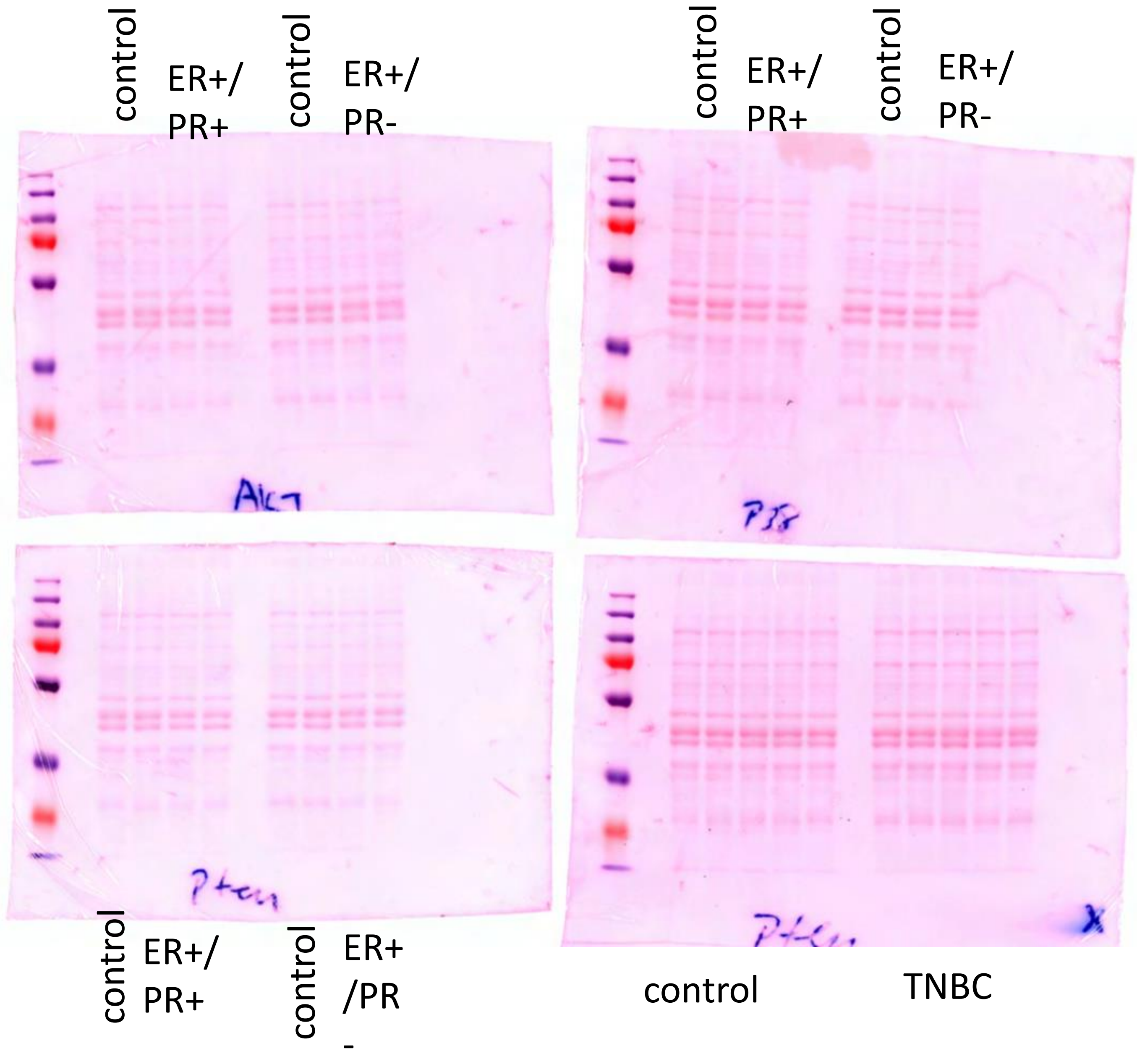
control
ER+/ PR+
control
ER+/ PR-
control
ER+/ PR+
control
ER+/ PR-
AKT
P38
Pten
Pten
control
ER+/ PR+
control
ER+ /PR -
control
TNBC

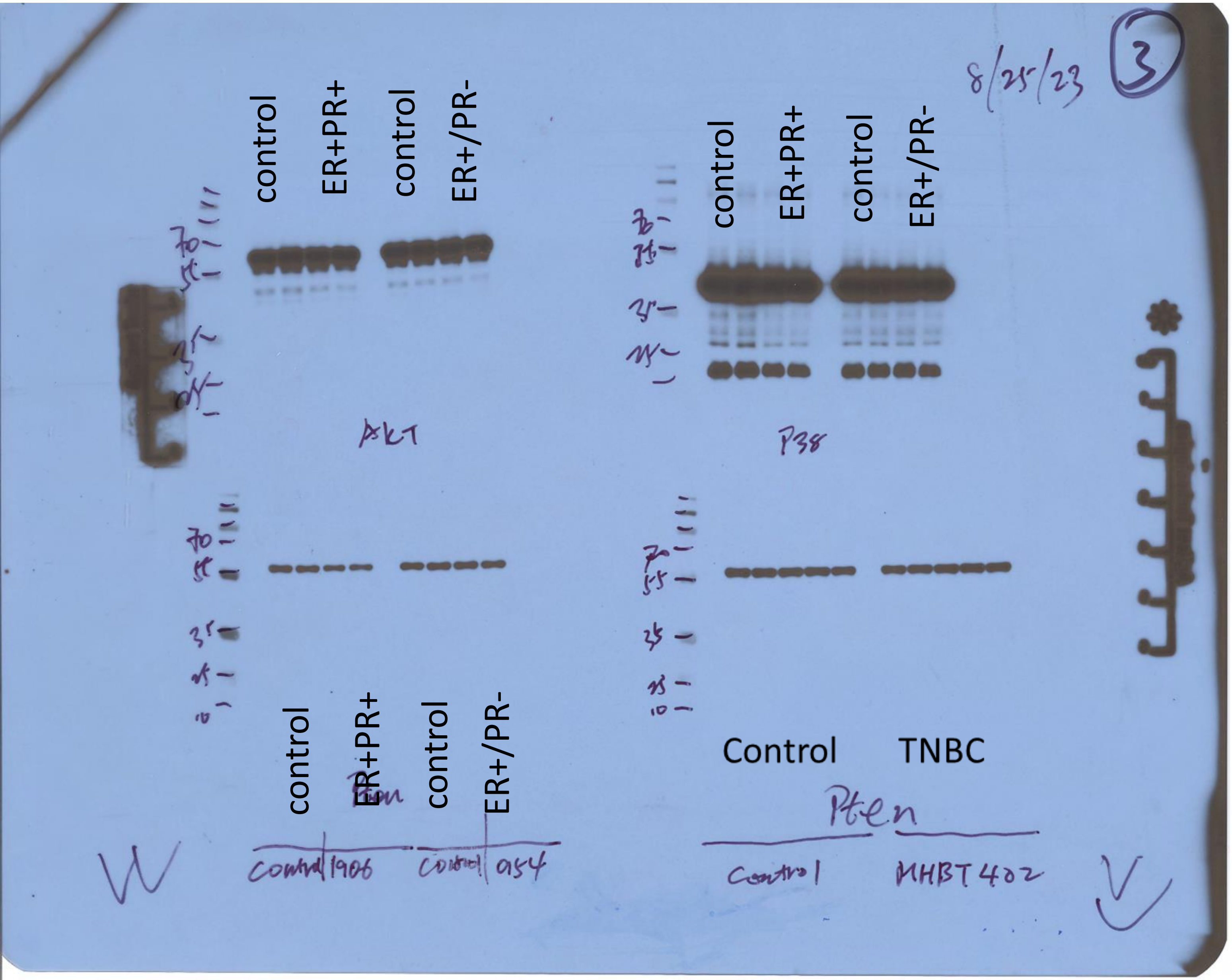
8/25/23
3
control
ER+PR+
control
ER+/PR-
AKT
control
ER+PR+
control
ER+/PR-
P38
control
ER+PR+
control
ER+/PR-
control 1906
control 954
Control
TNBC
Pten
Control
MHBT402

Raw data for PDX mice of ER+/PR+ and ER+/PR-

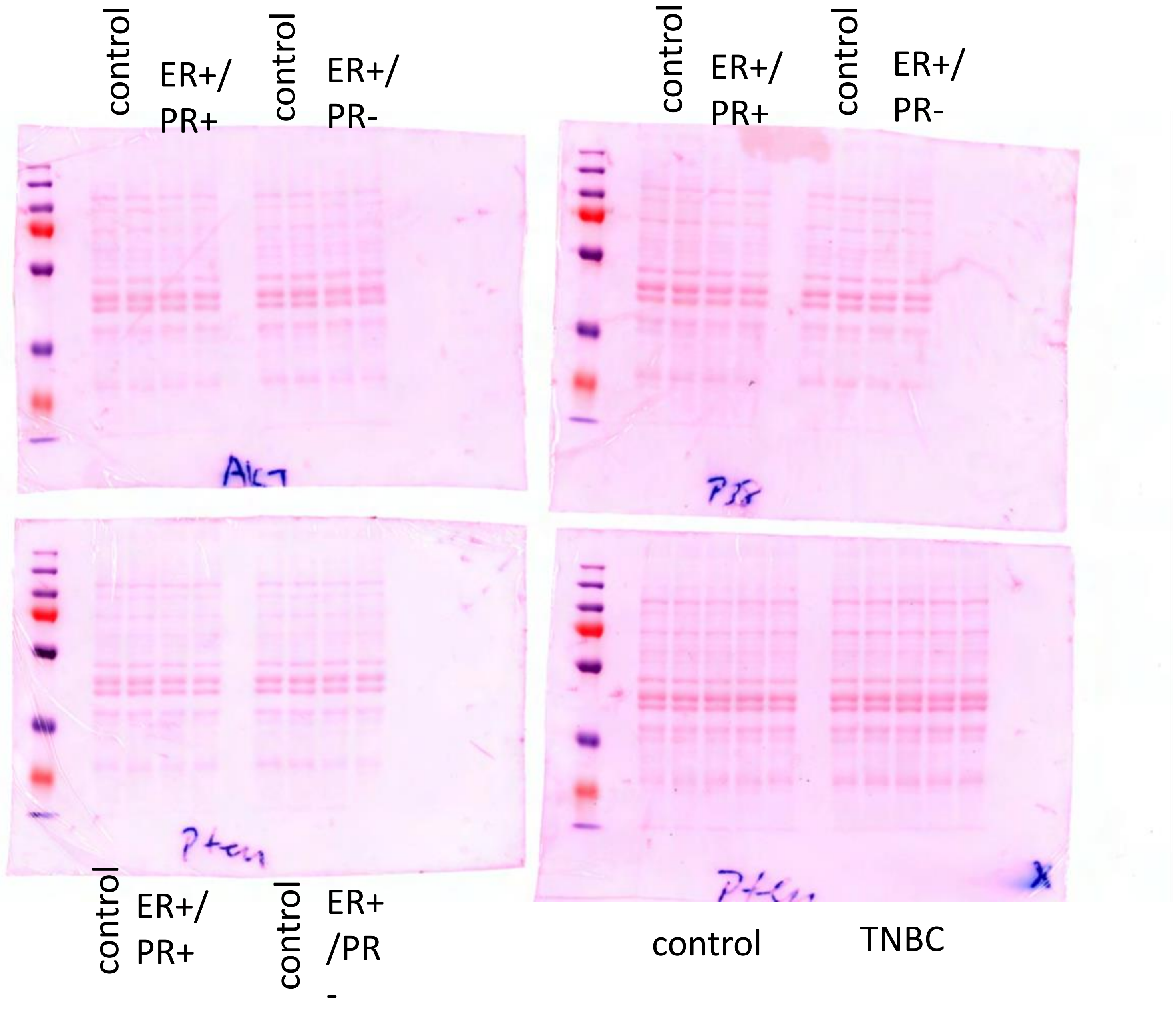
control
ER+/PR+
control
ER+/PR-
control
ER+/PR+
control
ER+/PR-
control
ER+/PR+
control
ER+
/PR
-
control
TNBC

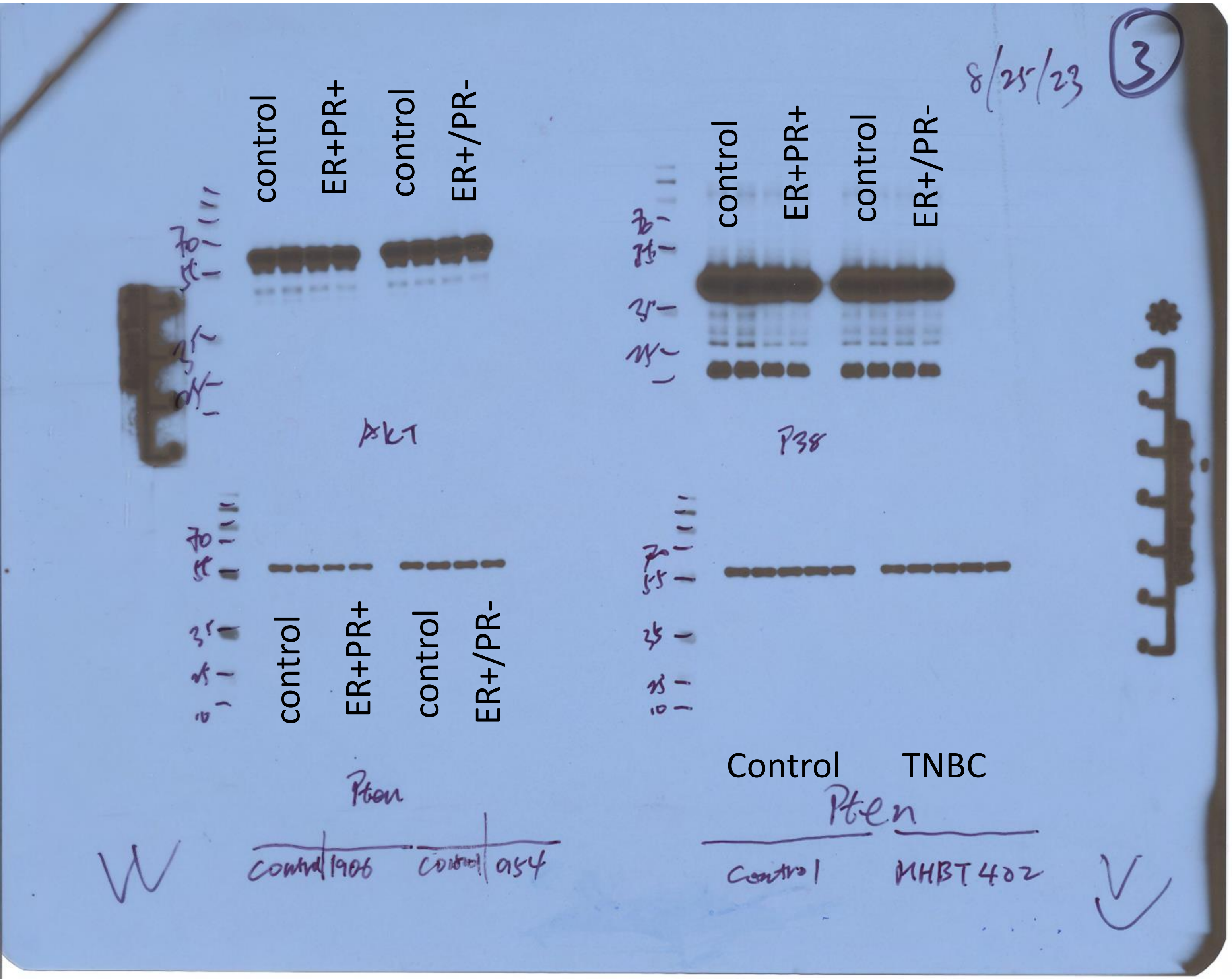

8/25/23
3
control
ER+PR+
control
ER+/PR-
control
ER+PR+
control
ER+/PR-
AKT
P38
control
ER+PR+
control
ER+/PR-
Pten
Control
TNBC
Pten
Control 1906
Control 954
Control
MHBT402

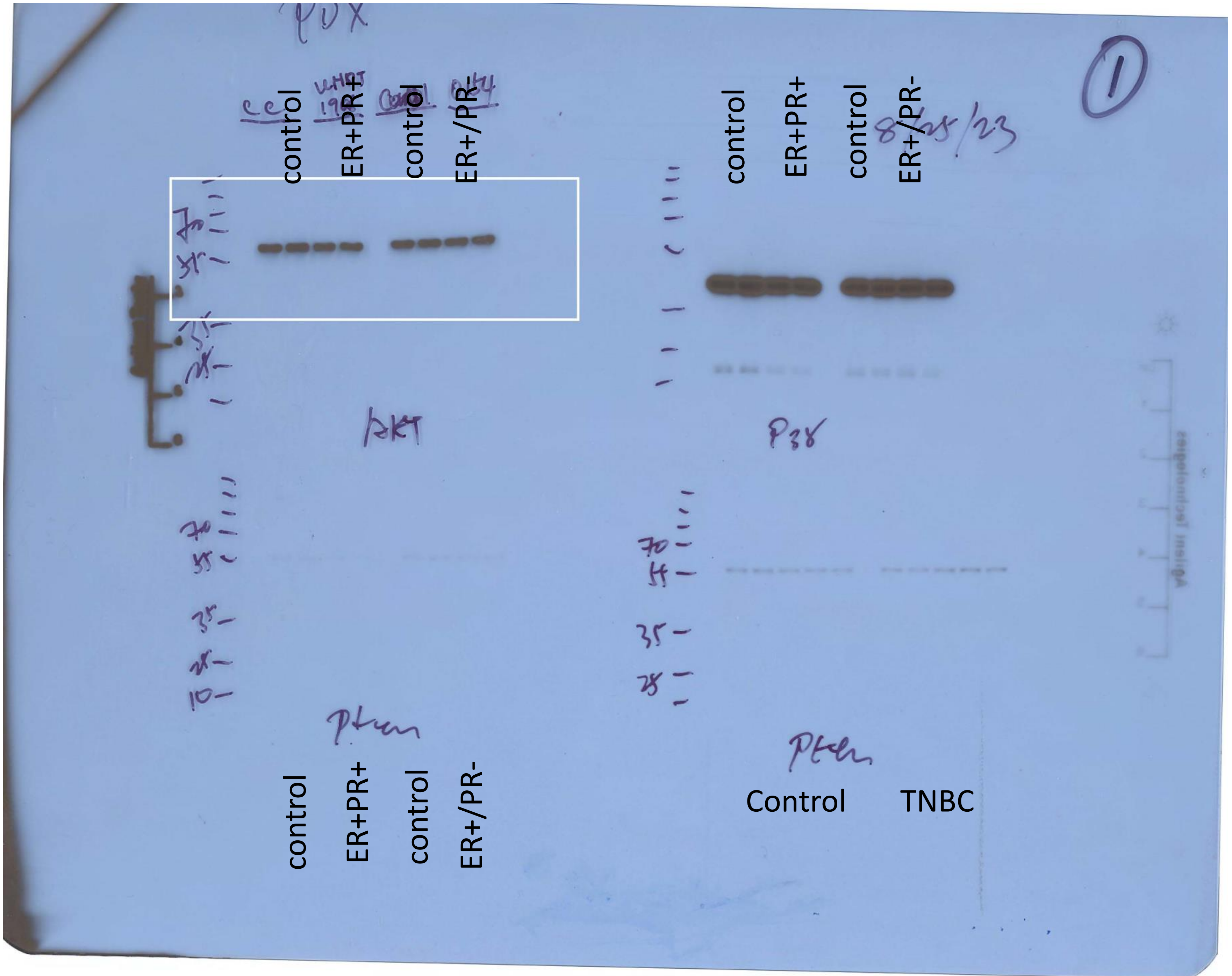
PDX
control
ER+PR+
control
ER+/PR-
control
ER+PR+
control
ER+/PR-
8/25/23
AKT
P38
Pten
Pten
control
ER+PR+
control
ER+/PR-
Control
TNBC

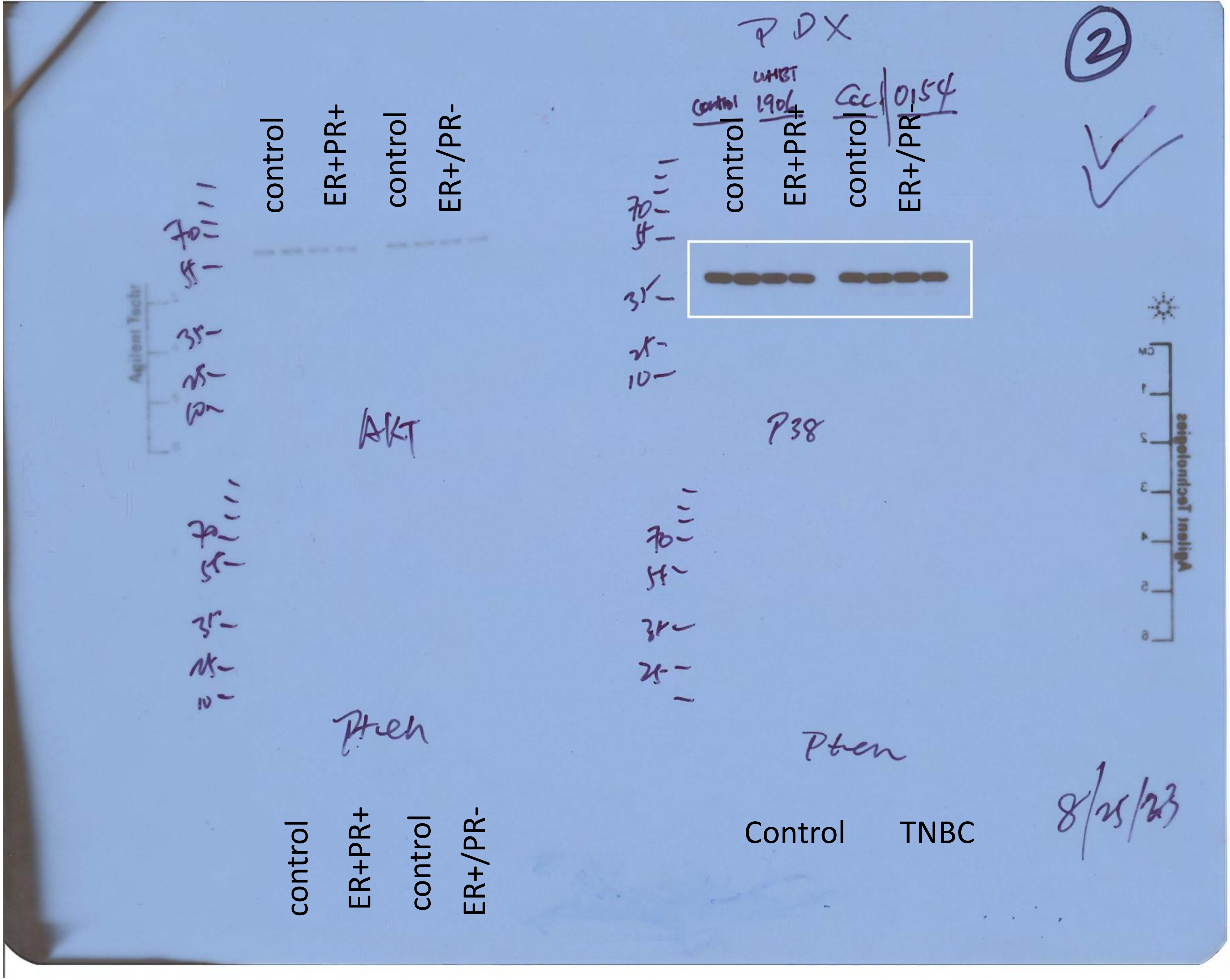

PDX
2
control
ER+PR+
control
ER+/PR-
control
WHBT 1906
ER+PR+
control
ER+/PR-
70
55
35
25
10
70
55
35
25
10
AKT
P38
70
55
35
25
10
70
55
35
25
Pten
Pten
control
ER+PR+
control
ER+/PR-
Control
TNBC
8/25/23

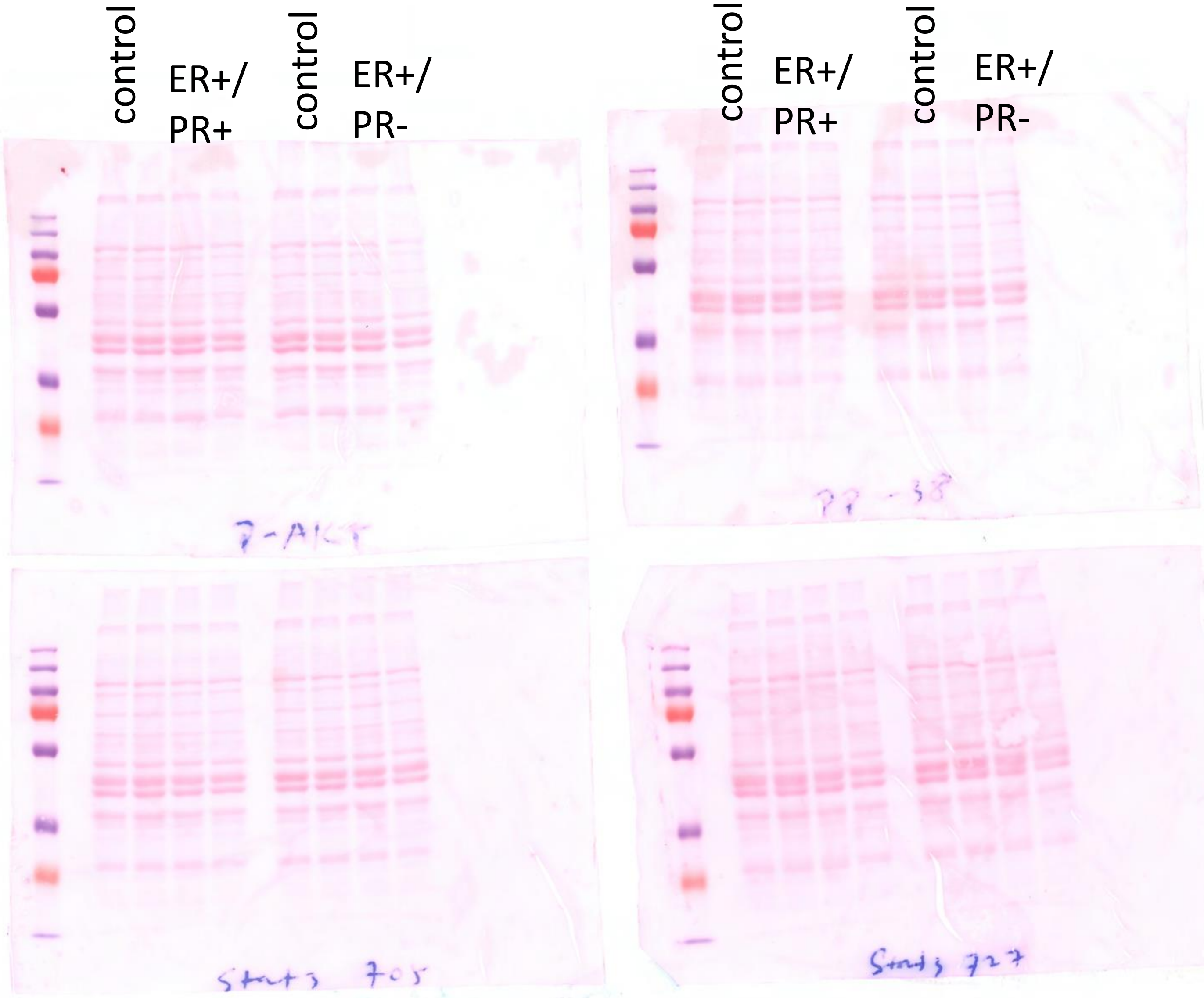
control
ER+/PR+
control
ER+/PR-
control
ER+/PR+
control
ER+/PR-
Stat3 705
Stat3 727

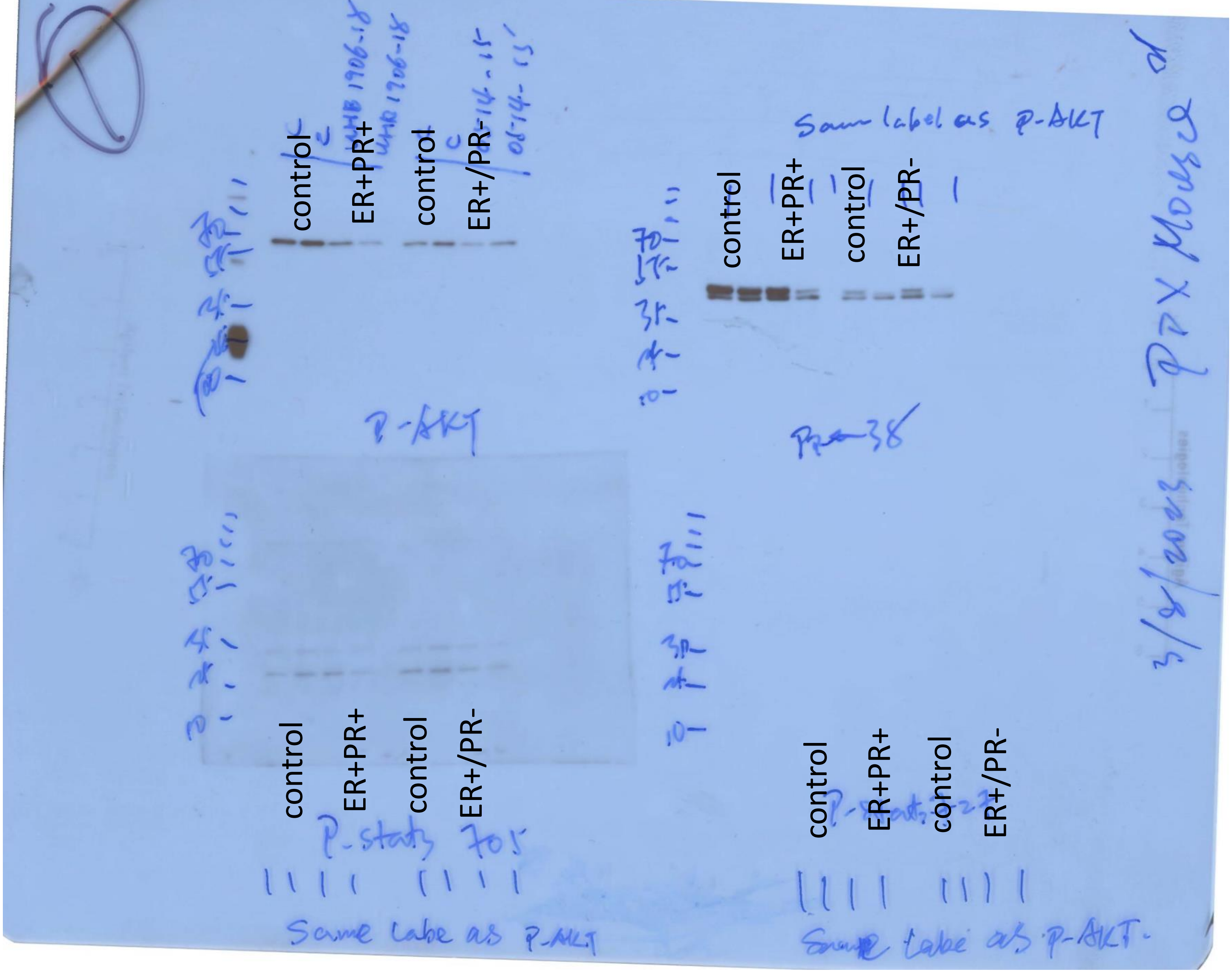
control
ER+PR+
control
ER+/PR-
P-AKT
Same label as P-AKT
control
ER+PR+
control
ER+/PR-
control
ER+PR+
control
ER+/PR-
P-stat3 705
Same label as P-AKT
control
ER+PR+
control
ER+/PR-
Same label as P-AKT
PDX Mouse 2

Raw data for Figure 5

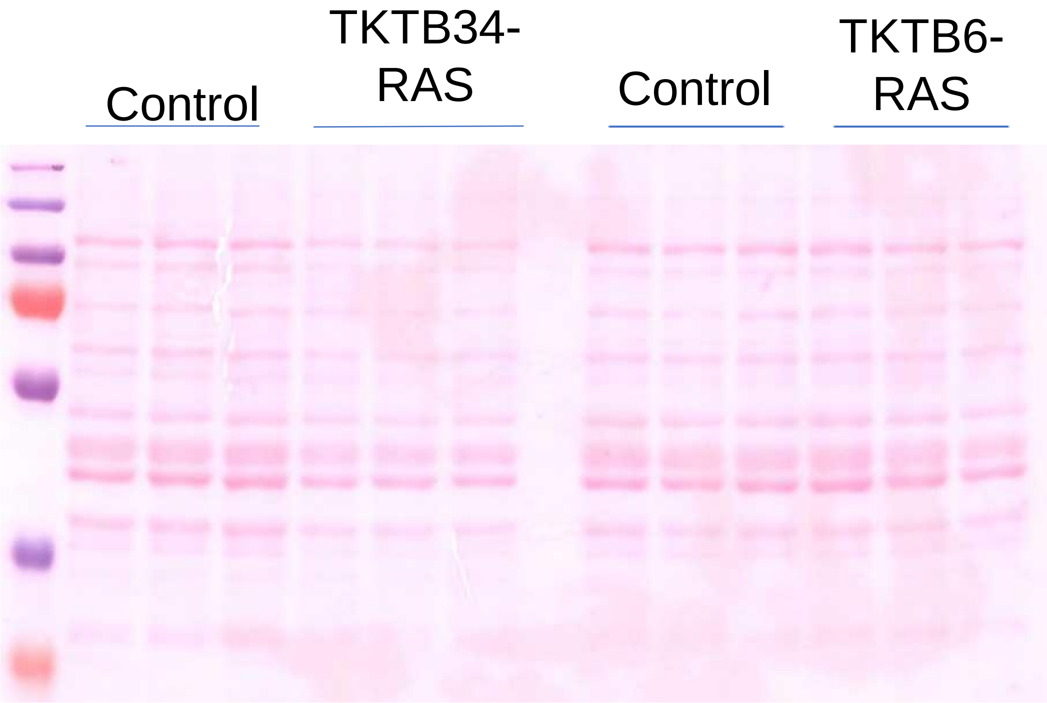
Control
TKTB34-
RAS
Control
TKTB6-
RAS

Ponceau s staining

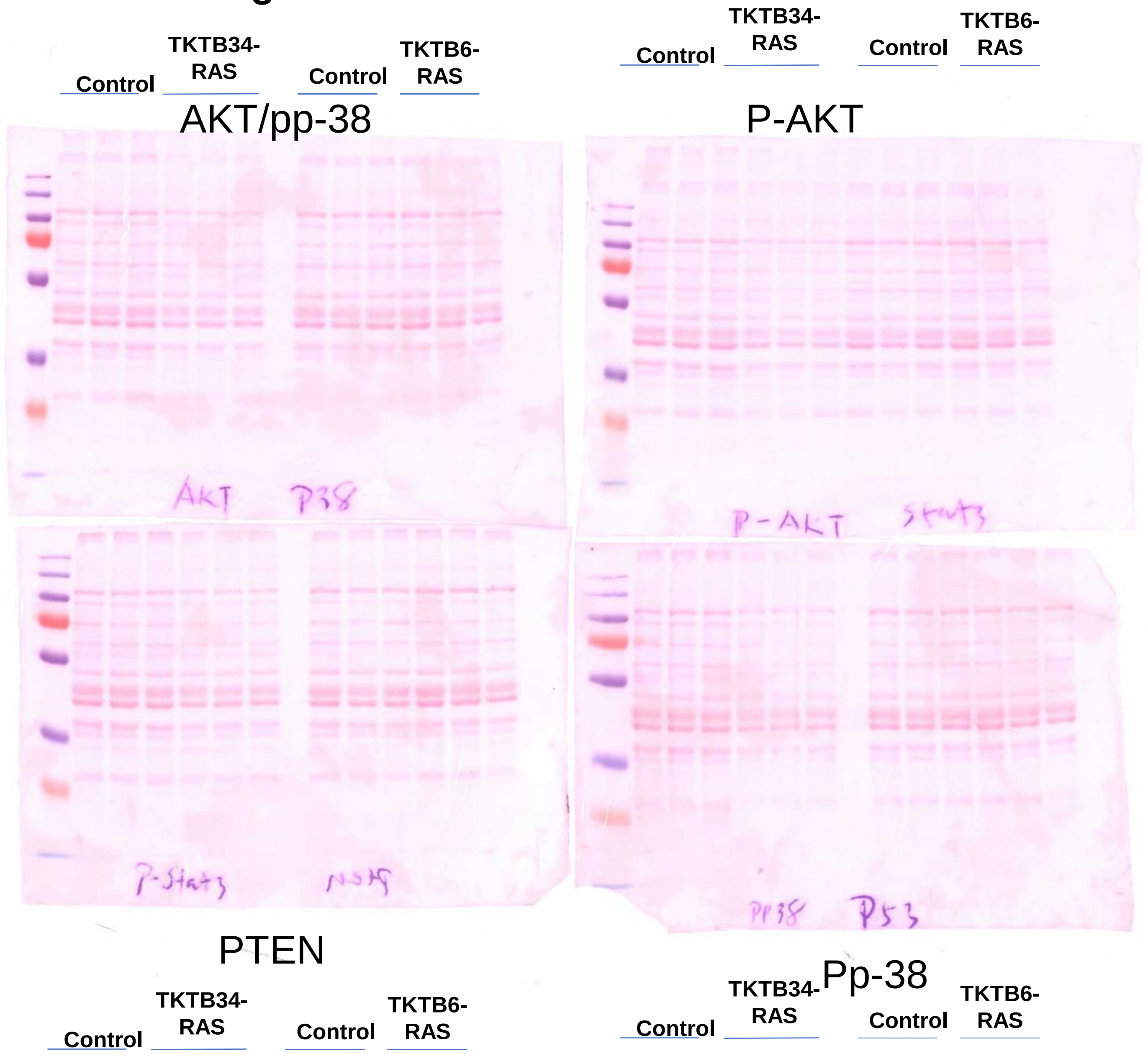

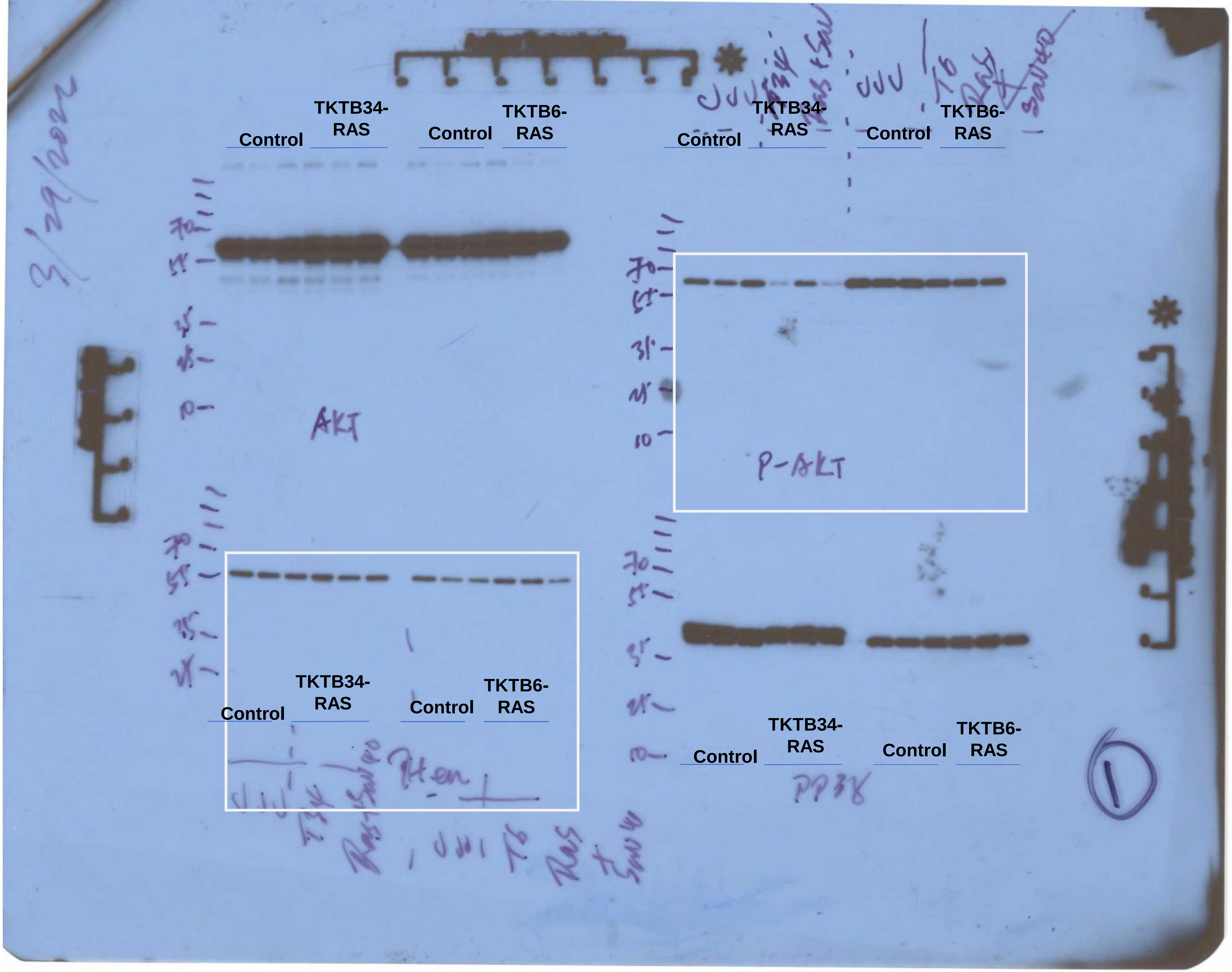
Control
TKTB34-RAS
Control
TKTB6-RAS
Control
TKTB34-RAS
Control
TKTB6-RAS
3/29/2022
AKT
P-AKT
Control
TKTB34-RAS
Control
TKTB6-RAS
Control
TKTB34-RAS
Control
TKTB6-RAS
PP38

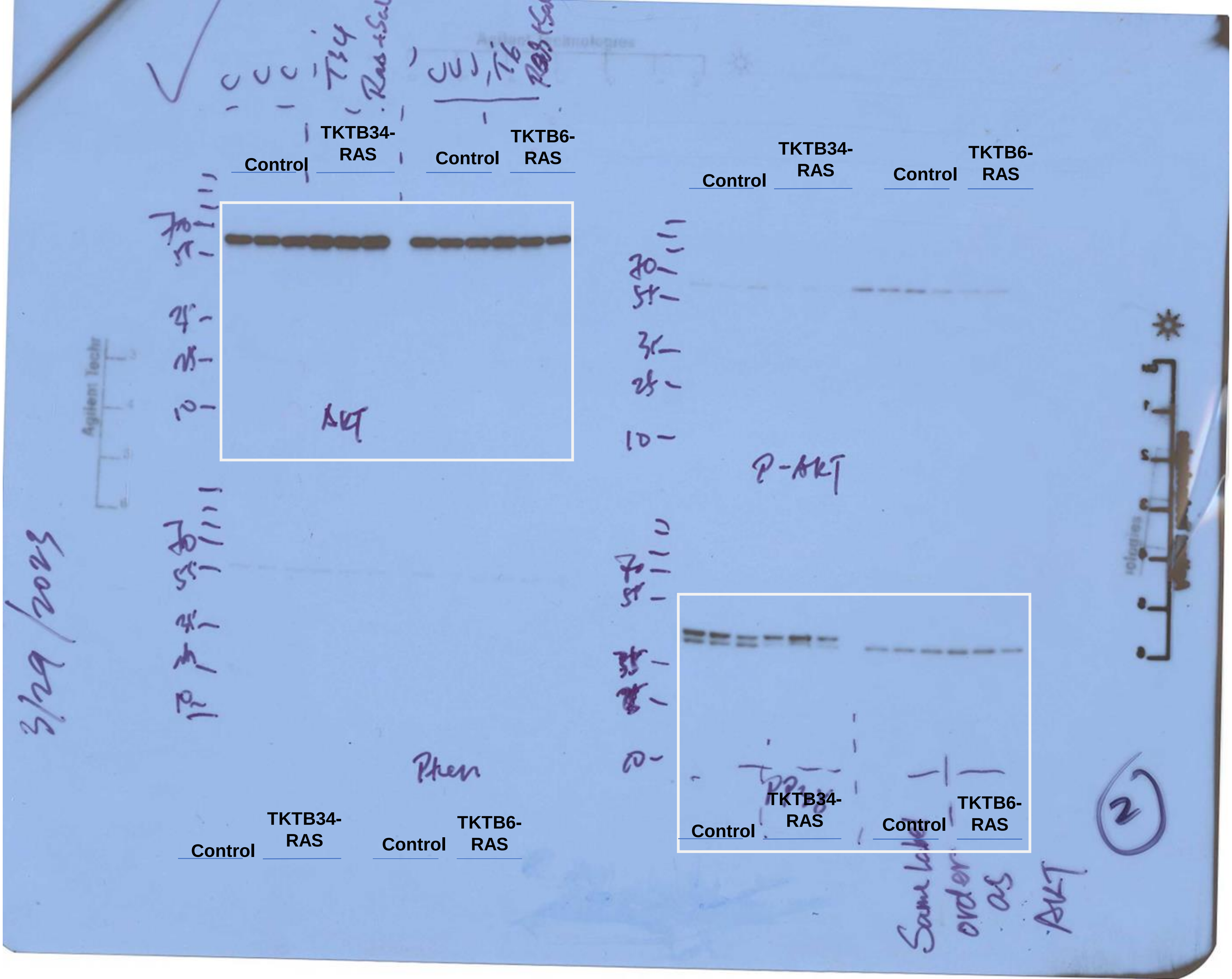
Control
TKTB34-RAS
Control
TKTB6-RAS
AKT
Control
TKTB34-RAS
Control
TKTB6-RAS
P-AKT
3/29/2023
Pten
Control
TKTB34-RAS
Control
TKTB6-RAS
Control
TKTB34-RAS
Control
TKTB6-RAS
Same lane order as AKT

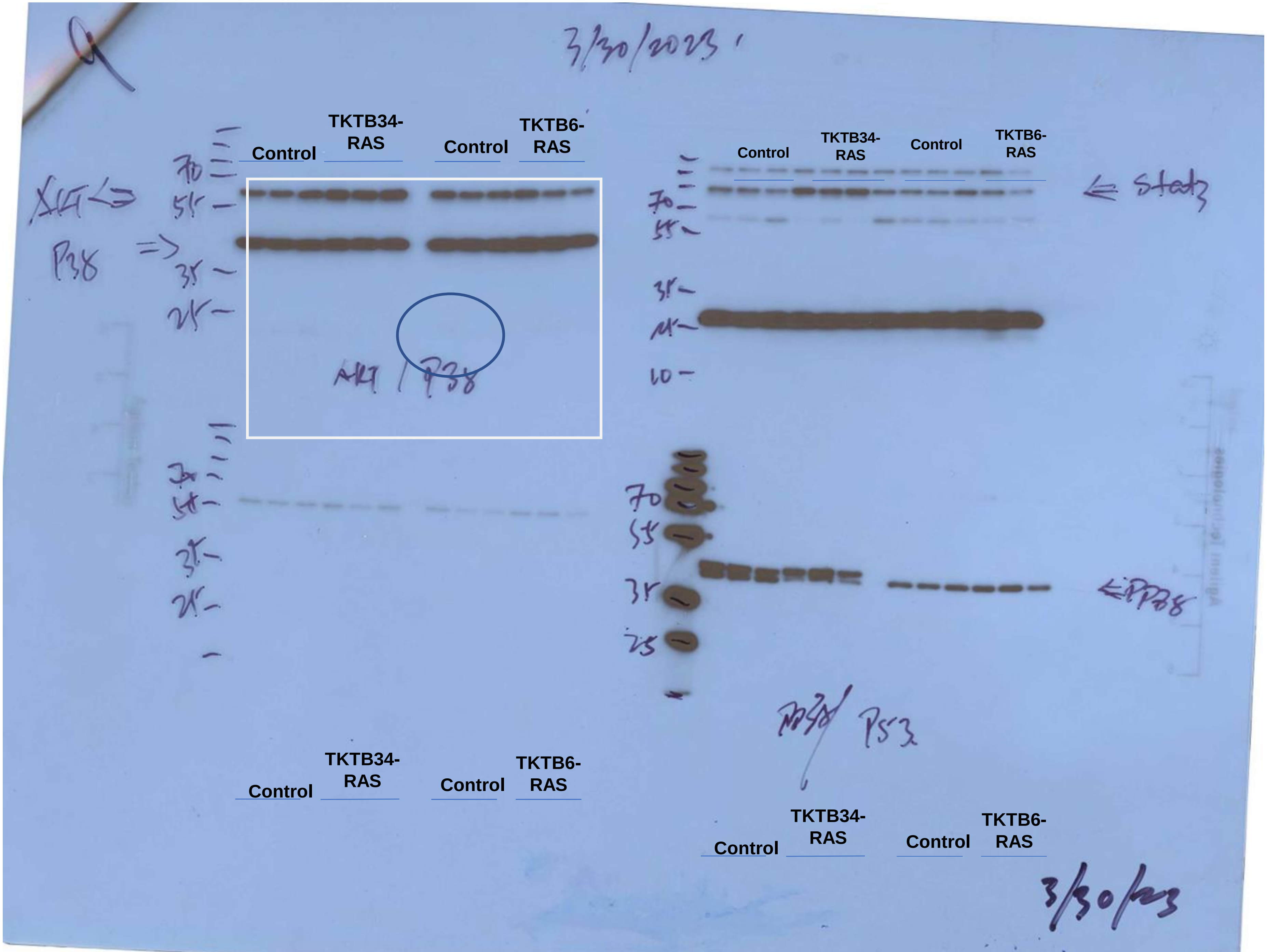
3/30/2023
Control
TKTB34-RAS
Control
TKTB6-RAS
70
55
35
25
AKT
P38
AKT / P38
Control
TKTB34-RAS
Control
TKTB6-RAS
Stat3
70
55
35
10
Control
TKTB34-RAS
Control
TKTB6-RAS
70
55
35
25
70
55
35
25
pP38
P53
Control
TKTB34-RAS
Control
TKTB6-RAS
3/30/23

TKTB34- PIK3CA raw data

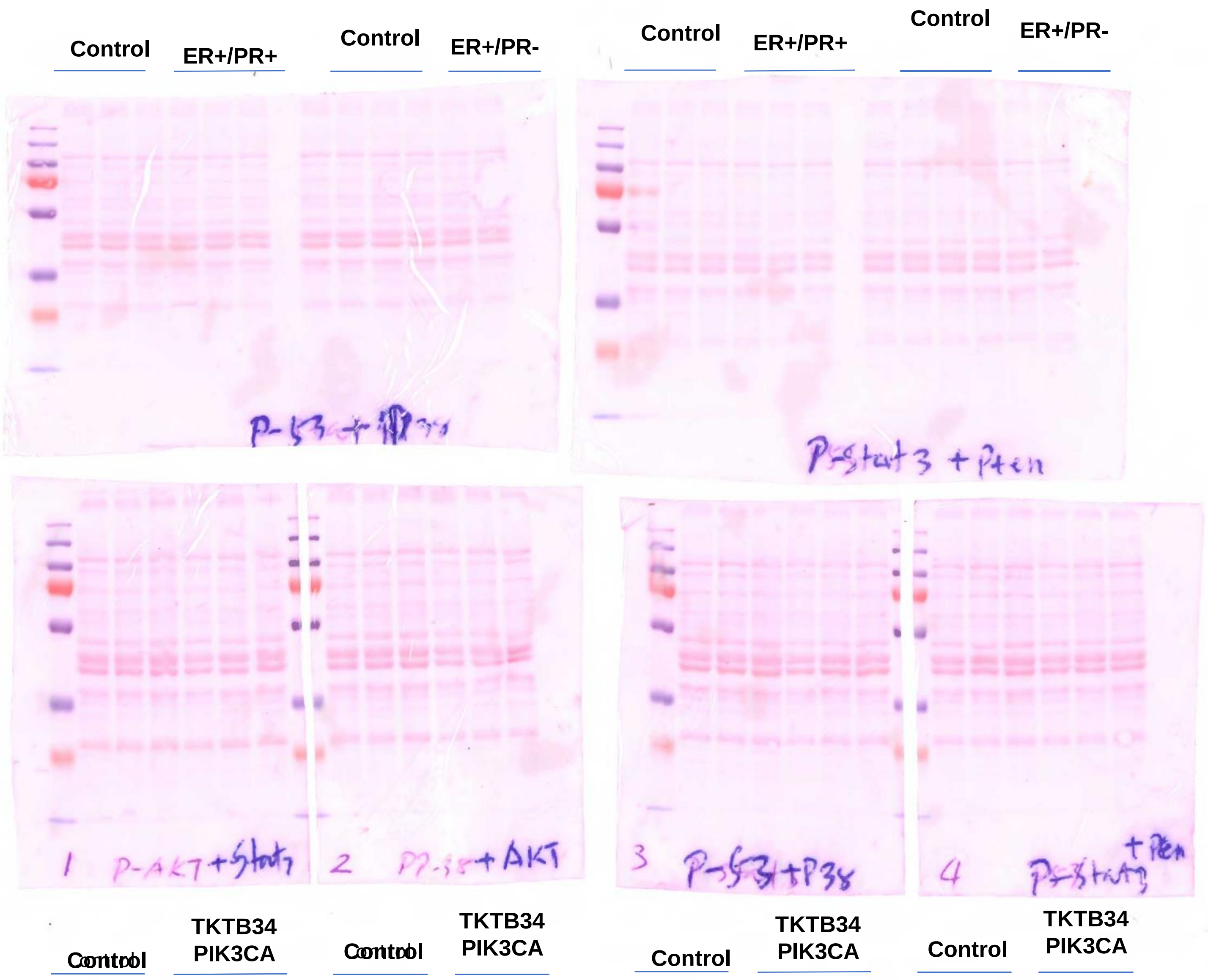
Control
ER+/PR+
Control
ER+/PR-
Control
ER+/PR+
Control
ER+/PR-
P-Stat3 + Pten
1 P-AKT+Stat3
2 P38 + AKT
3 P-53 + P38
4 + Pten
Control
TKTB34 PIK3CA
Control
TKTB34 PIK3CA
Control
TKTB34 PIK3CA
Control
TKTB34 PIK3CA

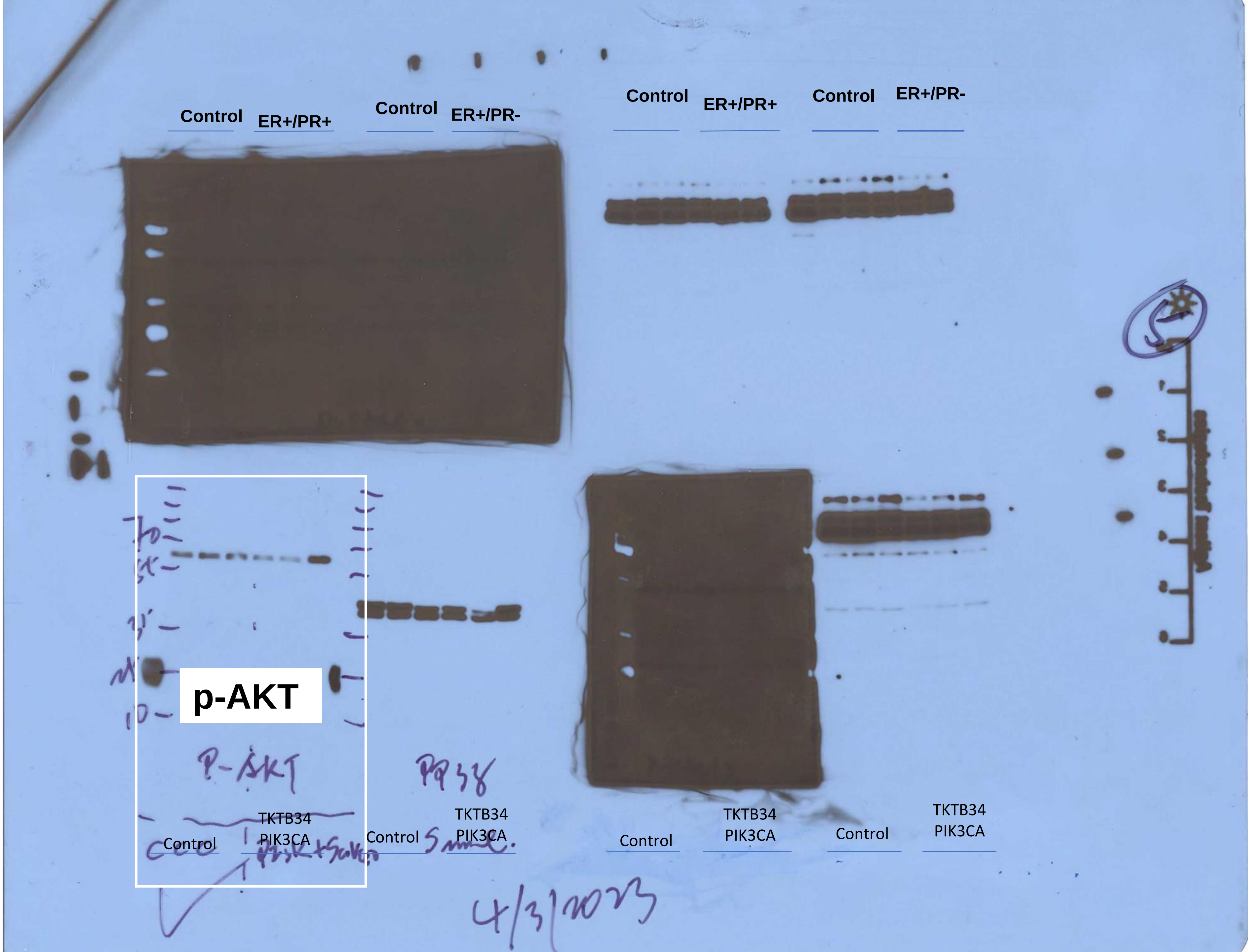
Control
ER+/PR+
Control
ER+/PR-
Control
ER+/PR+
Control
ER+/PR-
p-AKT
Control
TKTB34 PIK3CA
Control
TKTB34 PIK3CA
Control
TKTB34 PIK3CA
Control
TKTB34 PIK3CA

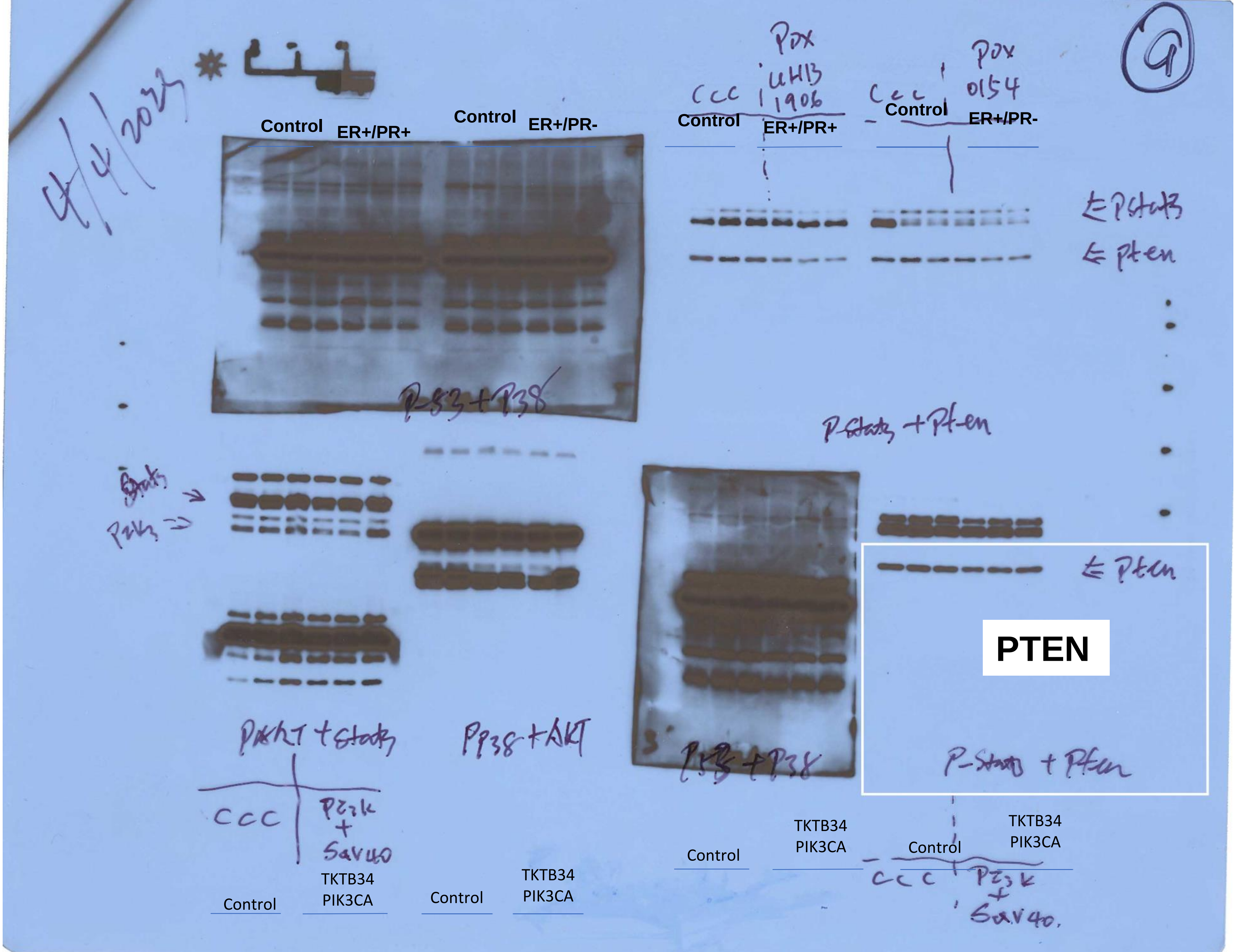
Control
ER+/PR+
Control
ER+/PR-
Control
ER+/PR+
Control
ER+/PR-
PTEN
Control
TKTB34
PIK3CA
Control
TKTB34
PIK3CA
Control
TKTB34
PIK3CA
Control
TKTB34
PIK3CA

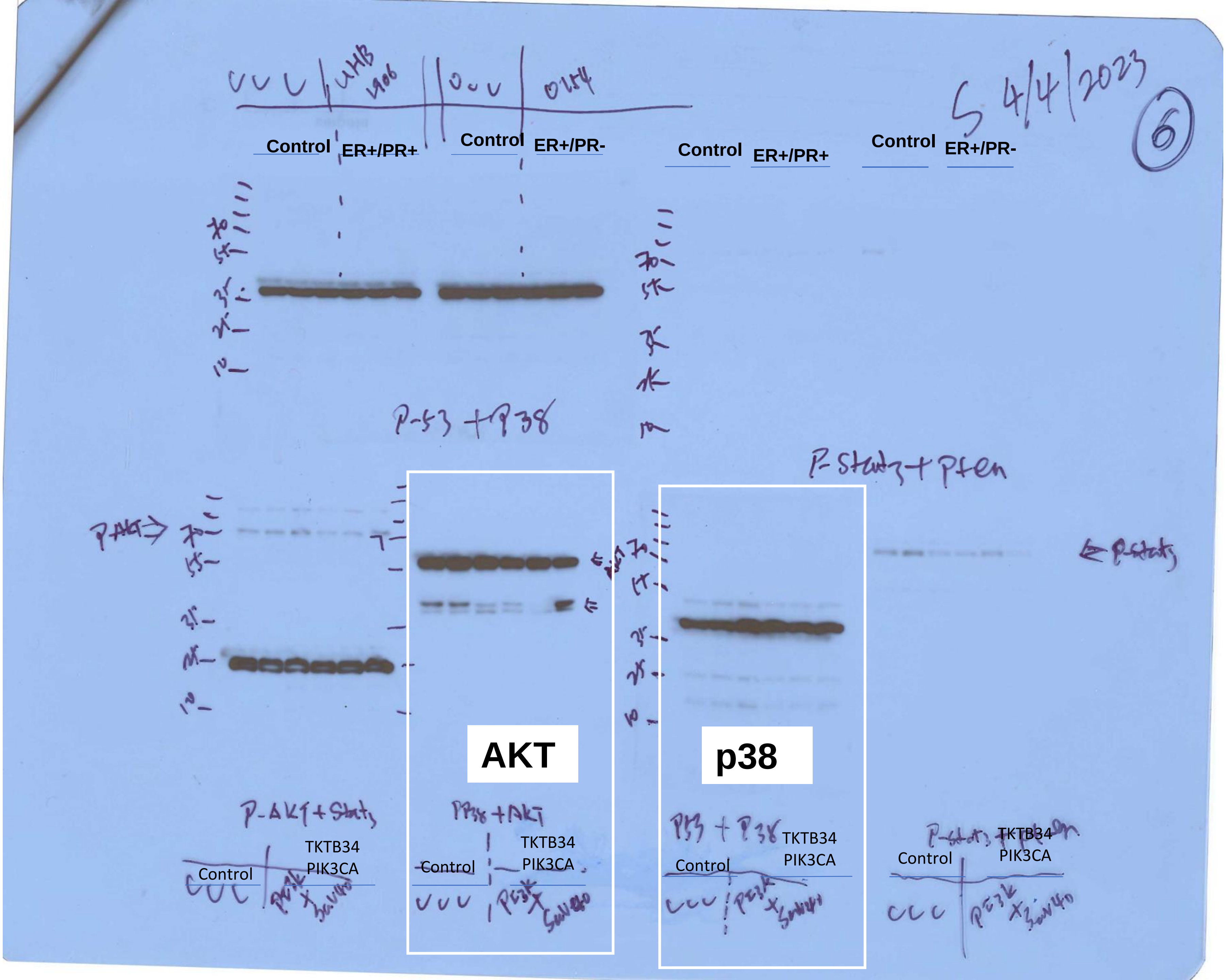
Control
ER+/PR+
Control
ER+/PR-
Control
ER+/PR+
Control
ER+/PR-
AKT
p38
Control
TKTB34
PIK3CA
Control
TKTB34
PIK3CA
Control
TKTB34
PIK3CA
Control
TKTB34
PIK3CA

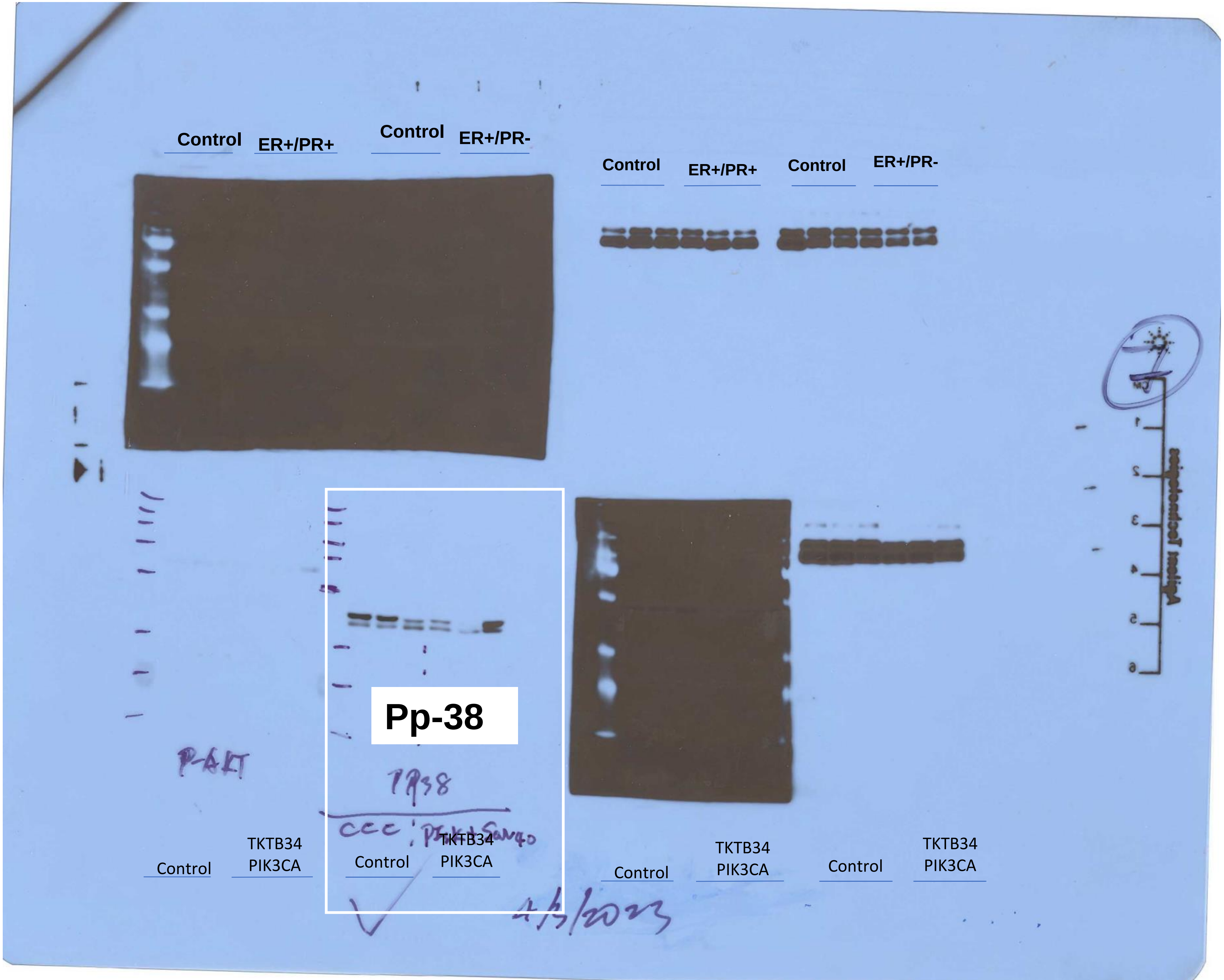

Control
ER+/PR+
Control
ER+/PR-
Control
ER+/PR+
Control
ER+/PR-
Pp-38
P-AKT
Control
TKTB34 PIK3CA
Control
TKTB34 PIK3CA
Control
TKTB34 PIK3CA
Control
TKTB34 PIK3CA

Raw data for Figure 6

Proteome Profiler Human XL Cytokine Array Kit ARY022B

Detects 109 human cytokine and chemokines simultaneously.

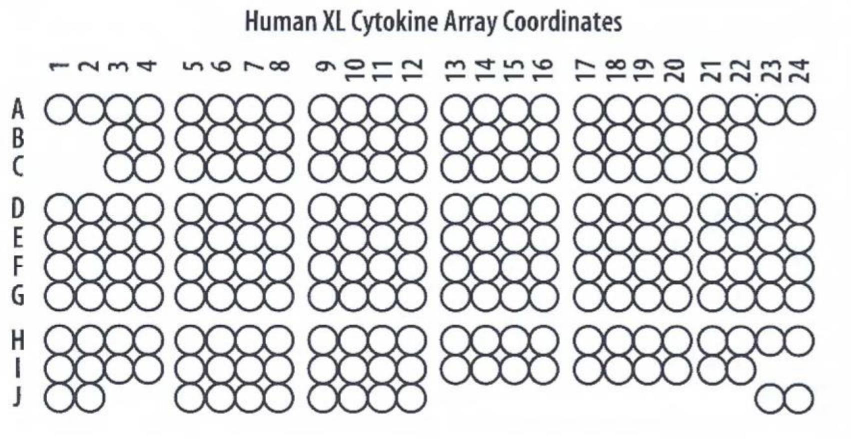
Human XL Cytokine Array Coordinates
1 2 3 4 5 6 7 8 9 10 11 12 13 14 15 16 17 18 19 20 21 22 23 24
A
B
C
D
E
F
G
H
I
J

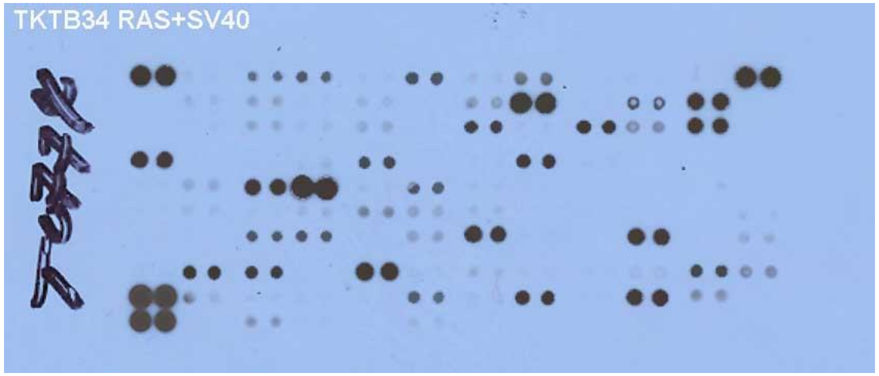
TKTB34 RAS+SV40

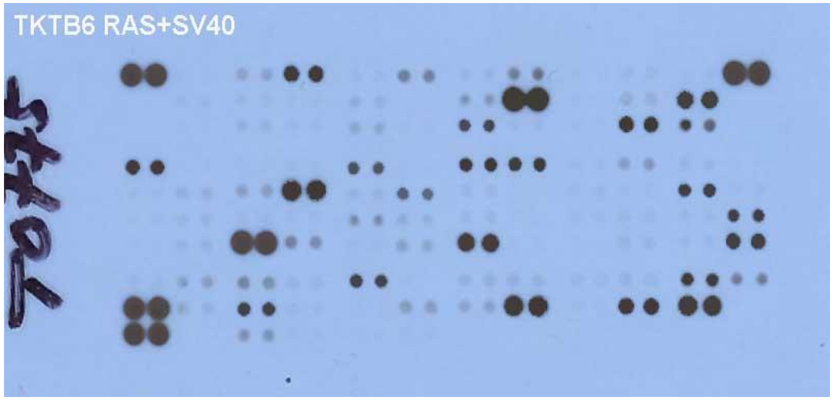
TKTB6 RAS+SV40

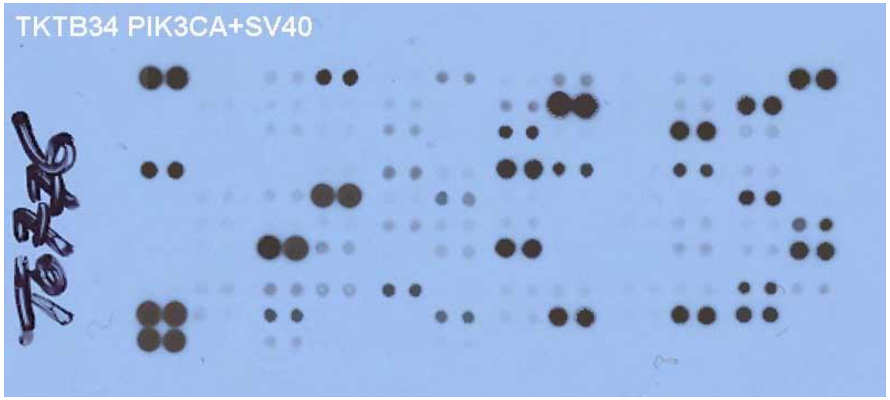
TKTB34 PIK3CA+SV40

APPENDIX

Refer to the table below for the Human XL Cytokine Array coordinates.

Coordinate	Analyte/Control	Entrez Gene ID	Alternate Nomenclature
A1, A2	Reference Spots	N/A	RS
A3, A4	Adiponectin	9370	Acrp30
A5, A6	Apolipoprotein A-I	335	ApoA1
A7, A8	Angiogenin	283	———
A9, A10	Angiopoietin-1	284	Ang-1, ANGPT1
A11, A12	Angiopoietin-2	285	Ang-2, ANGPT2
A13, A14	BAFF	10673	BLyS, TNFSF13B
A15, A16	BDNF	627	Brain-derived Neurotrophic Factor
A17, A18	Complement Component C5/C5a	727	C5/C5a
A19, A20	CD14	929	———
A21, A22	CD30	943	TNFRSF8
A23, A24	Reference Spots	N/A	RS
B3, B4	CD40 ligand	959	CD40L, TNFSF5, CD154, TRAP
B5, B6	Chitinase 3-like 1	1116	CHI3L1, YKL-40
B7, B8	Complement Factor D	1675	Adipsin, CFD
B9, B10	C-Reactive Protein	1401	CRP
B11, B12	Cripto-1	6997	Teratocarcinoma-derived Growth Factor
B13, B14	Cystatin C	1471	CST3, ARMD11
B15, B16	Dkk-1	22943	Dickkopf-1
B17, B18	DPPIV	1803	CD26, DPP4, Dipeptidyl-peptidase IV
B19, B20	EGF	1950	Epidermal Growth Factor
B21, B22	EMMPRIN	682	CD147, Basigin
C3, C4	ENA-78	6374	CXCL5
C5, C6	Endoglin	2022	CD105, ENG
C7, C8	Fas Ligand	356	TNFSF6, CD178, CD95L
C9, C10	FGF basic	2247	FGF-2
C11, C12	FGF-7	2252	KGF
C13, C14	FGF-19	9965	———
C15, C16	Flt-3 Ligand	2323	FLT3LG
C17, C18	G-CSF	1440	CSF3
C19, C20	GDF-15	9518	MIC-1
C21, C22	GM-CSF	1437	CSF2
D1, D2	GROα	2919	CXCL1, MSGA-α
D3, D4	Growth Hormone	2688	GH, Somatotropin
D5, D6	HGF	3082	Scatter Factor, SF
D7, D8	ICAM-1	3383	CD54
D9, D10	IFN-γ	3458	IFNG
D11, D12	IGFBP-2	3485	———

APPENDIX *CONTINUED*

Coordinate	Analyte/Control	Entrez Gene ID	Alternate Nomenclature
D13, D14	IGFBP-3	3486	———
D15, D16	IL-1α	3552	IL-1F1
D17, D18	IL-1β	3553	IL-1F2
D19, D20	IL-1ra	3557	IL-1F3
D21, D22	IL-2	3558	———
D23, D24	IL-3	3562	———
E1, E2	IL-4	3565	———
E3, E4	IL-5	3567	———
E5, E6	IL-6	3569	———
E7, E8	IL-8	3576	CXCL8
E9, E10	IL-10	3586	———
E11, E12	IL-11	3589	———
E13, E14	IL-12 p70	3593	———
E15, E16	IL-13	3596	———
E17, E18	IL-15	3600	———
E19, E20	IL-16	3603	———
E21, E22	IL-17A	3605	IL-17, CTLA8
E23, E24	IL-18 Bpa	10068	———
F1, F2	IL-19	29949	———
F3, F4	IL-22	50616	IL-TIF
F5, F6	IL-23	51561	IL-23A, SGRF
F7, F8	IL-24	11009	C49A, FISP, MDA-7, MOB-5, ST16
F9, F10	IL-27	246778	———
F11, F12	IL-31	386653	———
F13, F14	IL-32	9235	———
F15, F16	IL-33	90865	C9orf26, DVS27, NF-HEV
F17, F18	IL-34	146433	C16orf77
F19, F20	IP-10	3627	CXCL10
F21, F22	I-TAC	6373	CXCL11, SCYB9B
F23, F24	Kallikrein 3	354	PSA, KLK3
G1, G2	Leptin	3952	OB
G3, G4	LIF	3976	———
G5, G6	Lipocalin-2	3934	NGAL, LCN2, Siderocalin
G7, G8	MCP-1	6347	CCL2, MCAF
G9, G10	MCP-3	6354	CCL7, MARC
G11, G12	M-CSF	1435	CSF1
G13, G14	MIF	4282	———
G15, G16	MIG	4283	CXCL9

APPENDIX *CONTINUED*

Coordinate	Analyte/Control	Entrez Gene ID	Alternate Nomenclature
G17, G18	MIP-1α/MIP-1β	6348/6351	CCL3/CCL4
G19, G20	MIP-3α	6364	CCL20, Exodus-1, LARC
G21, G22	MIP-3β	6363	CCL19, ELC
G23, G24	MMP-9	4318	CLG4B, Gelatinase B
H1, H2	Myeloperoxidase	4353	MPO, Lactoperoxidase
H3, H4	Osteopontin	6696	OPN
H5, H6	PDGF-AA	5154	———
H7, H8	PDGF-AB/BB	5154/5155	———
H9, H10	Pentraxin 3	5806	PTX3, TSG-14
H11, H12	PF4	5196	CXCL4
H13, H14	RAGE	177	———
H15, H16	RANTES	6352	CCL5
H17, H18	RBP-4	5950	———
H19, H20	Relaxin-2	6019	RLN2, RLXH2
H21, H22	Resistin	56729	ADSF, FIZZ3, RETN
H23, H24	SDF-1α	6387	CXCL12, PBSF
I1, I2	Serpin E1	5054	PAI-I, PAI-1, Nexin
I3, I4	SHBG	6462	ABP
I5, I6	ST2	9173	IL-1 R4, IL1RL1, ST2L
I7, I8	TARC	6361	CCL17
I9, I10	TFF3	7033	ITF, TFI
I11, I12	TfR	7037	CD71, TFR1, TFRC, TRFR
I13, I14	TGF-α	7039	TGFA
I15, I16	Thrombospondin-1	7057	THBS1, TSP-1
I17, I18	TNF-α	7124	TNFSF1A
I19, I20	uPAR	5329	PLAUR
I21, I22	VEGF	7422	BEGFA
J1, J2	Reference Spots	N/A	RS
J5, J6	Vitamin D BP	2638	VDB, DBP, VDBP
J7, J8	CD31	5175	PECAM-1
J9, J10	TIM-3	84868	HAVCR2
J11, J12	VCAM-1	7412	CD106
J23, J24	Negative Controls	N/A	Control (-)

First test

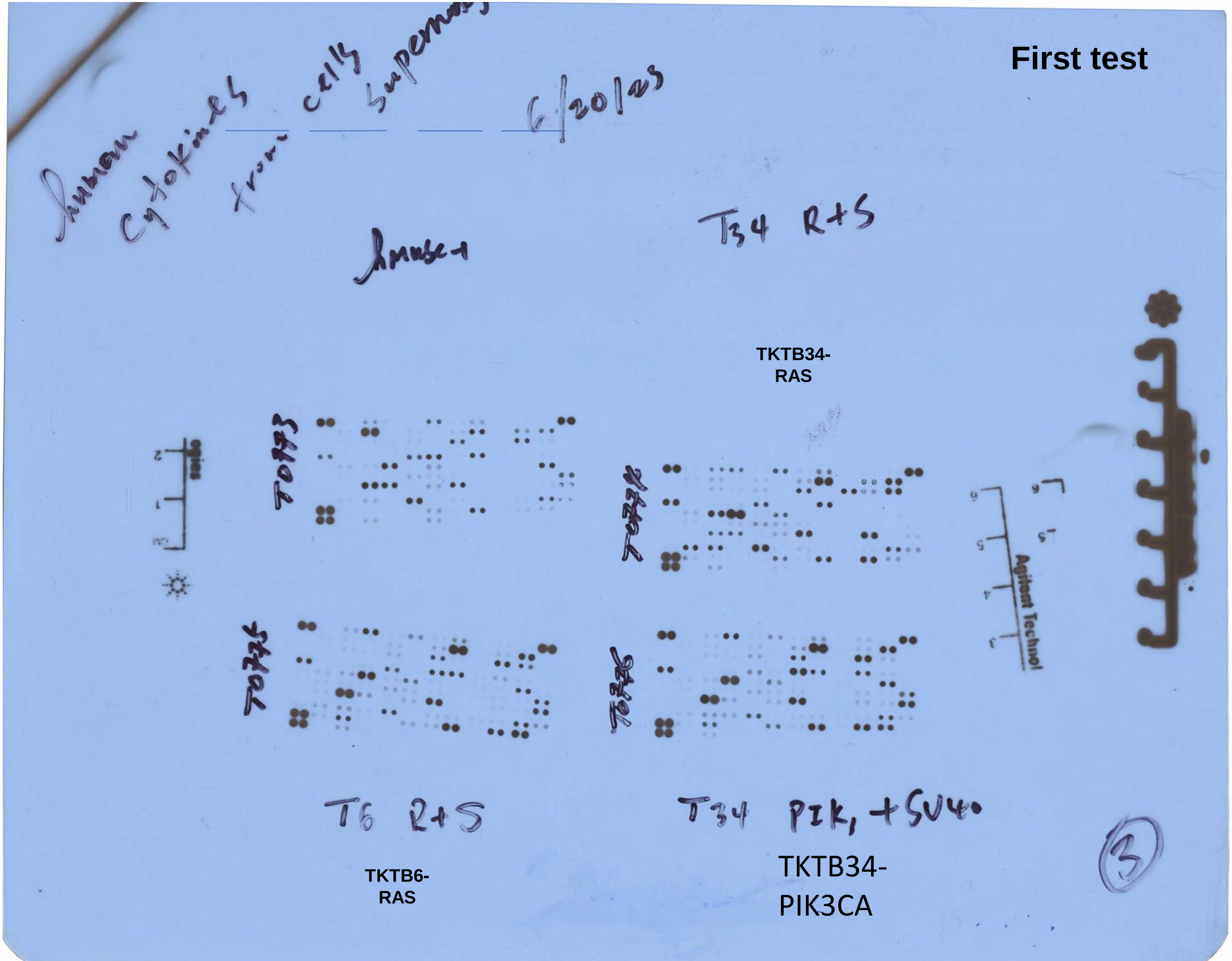

First test
human cytokines from cells supernatant
6/20/23
T34 R+S
TKTB34-RAS
T6 R+S
TKTB6-RAS
T34 PIK, +SV40
TKTB34-PIK3CA
Agilent Technol

Second test

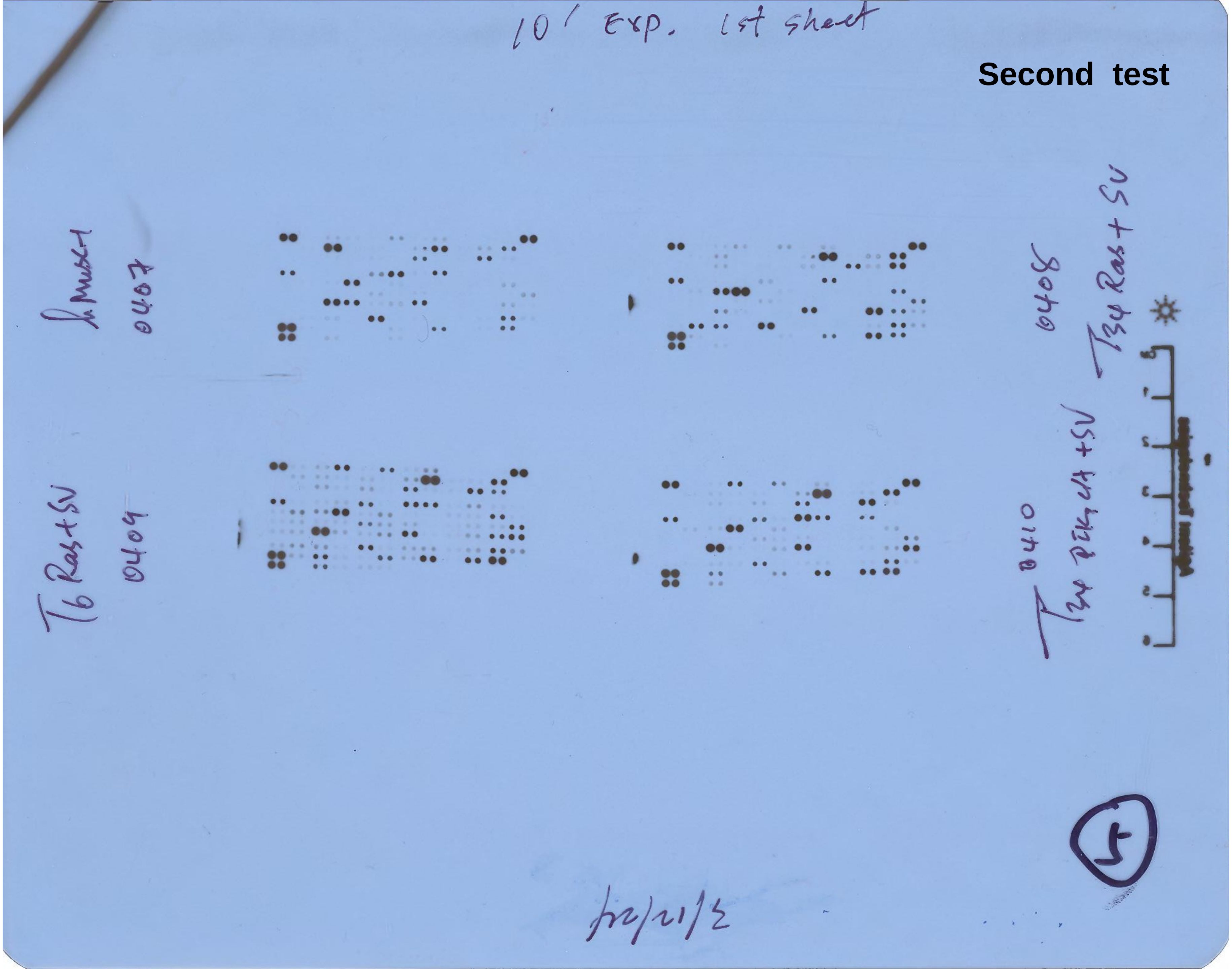
10' Exp. 1st sheet
0407
0408
T34 Ras + SV
T6 Ras+SV
0409
0410
T34 PIK3CA +SV
5
3/12/14
