# Supplementary figures and images for: Mutant RAS-driven Secretome Causes Skeletal Muscle Defects in Breast Cancer
Source: Cancer Res Commun. 2024 May 15;4(5):1282–95. doi: 10.1158/2767-9764.CRC-24-0045 (PMC11094532; doi:10.1158/2767-9764.CRC-24-0045)

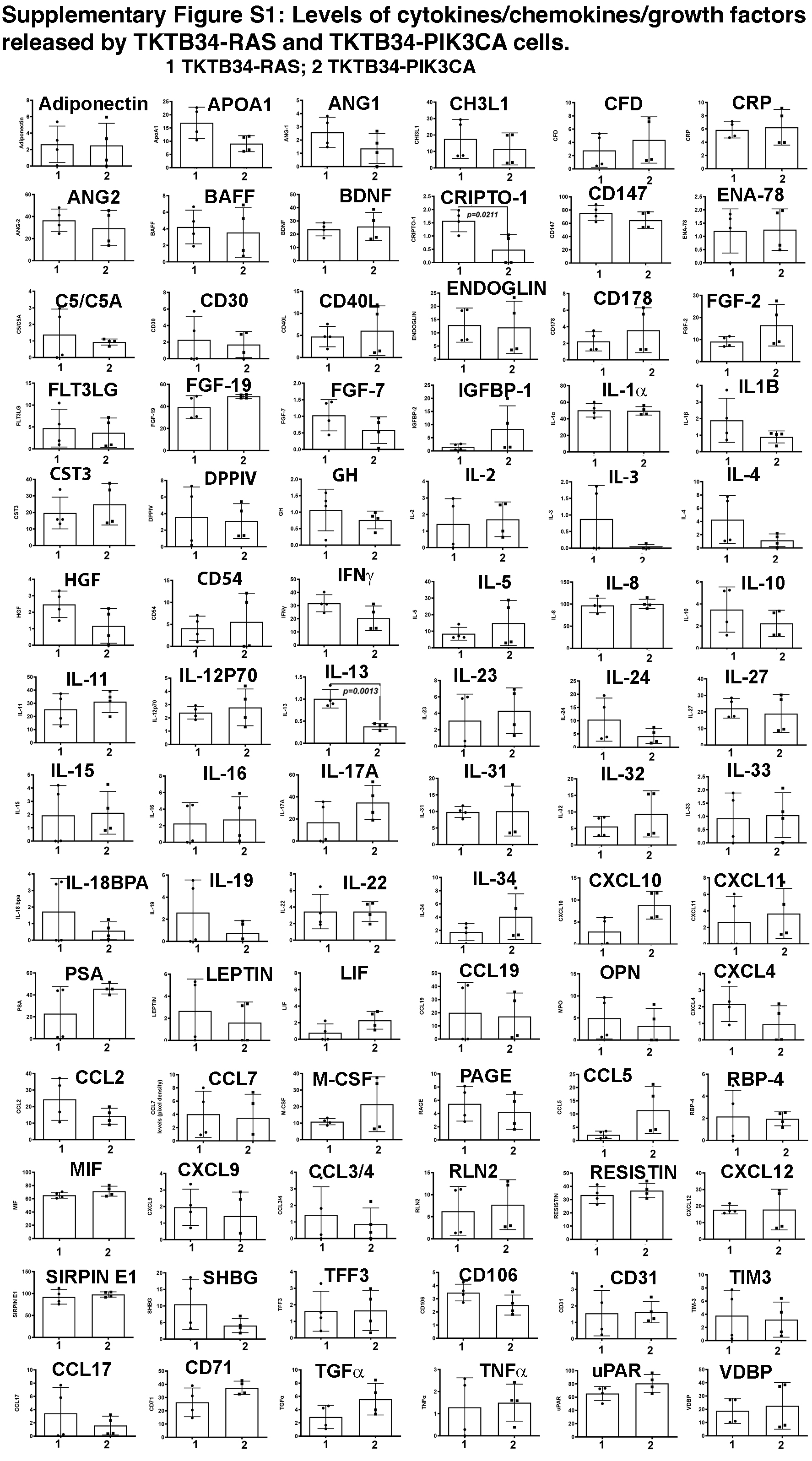

Supplement: Supplementary Figure S1 — Shows the levels of cytokines/chemokines/growth factors released by TKTB34-RAS and TKTB34-PIK3CA cells. Related to Figure 6 of the main manuscript. [file crc-24-0045-s03.jpeg]
